# Antiaromatic expanded porphyrin enables redox-tunable photoconductivity in columnar liquid crystals

**DOI:** 10.1039/d6sc06405a

**Published:** 2026-09-14

**Authors:** Duong D. Nguyen, W. Ryan Osterloh, Alberto Concellón, Teresa Sierra, Jorge Labella, Shu Seki, Jonathan L. Sessler

**Affiliations:** a Department of Chemistry, The University of Texas at Austin 105 East 24th Street Austin Texas 78712-1224 USA sessler@cm.utexas.edu; b Department of Chemical Science and Engineering, Graduate School of Engineering, Kyoto University Nishikyo-ku Kyoto 615-8510 Japan labella.jorge.54u@st.kyoto-u.ac.jp seki@moleng.kyoto-u.ac.jp; c Instituto de Nanociencia y Materiales de Aragón (INMA), Departamento de Química Orgánica, Facultad de Ciencias, CSIC-Universidad de Zaragoza Zaragoza 50009 Spain tsierra@unizar.es

## Abstract

Liquid crystals (LCs) based on π-conjugated building blocks are well-established platforms for engineering soft optoelectronic materials. However, the incorporation of antiaromatic frameworks-despite their potential to unlock new functional paradigms-remains highly challenging. Herein, we report an antiaromatic expanded porphyrin that upon heating and then cooling self-assembles into a highly ordered rectangular columnar mesophase (Col_r_). The resulting supramolecular organization enables photoinduced charge transport in the LC state that is markedly enhanced compared to the amorphous precursor state. Reductive (n-type) and oxidative (p-type) chemical doping was found to generate stabilized open-shell radical species within the Col_r_ assemblies and further boost their photoconductivity. The findings reported here establish antiaromatic expanded porphyrins as promising platforms for the construction of redox-responsive semiconducting soft materials.

## Abstract 2

An antiaromatic expanded porphyrin self-assembles into a rectangular columnar liquid crystal. This enables intrinsic photoconductivity, reaching *ϕ*Σ*µ* values up to 5.8 × 10^−4^ cm^2^ V^−1^ s^−1^ upon doping.

## Graphical abstract

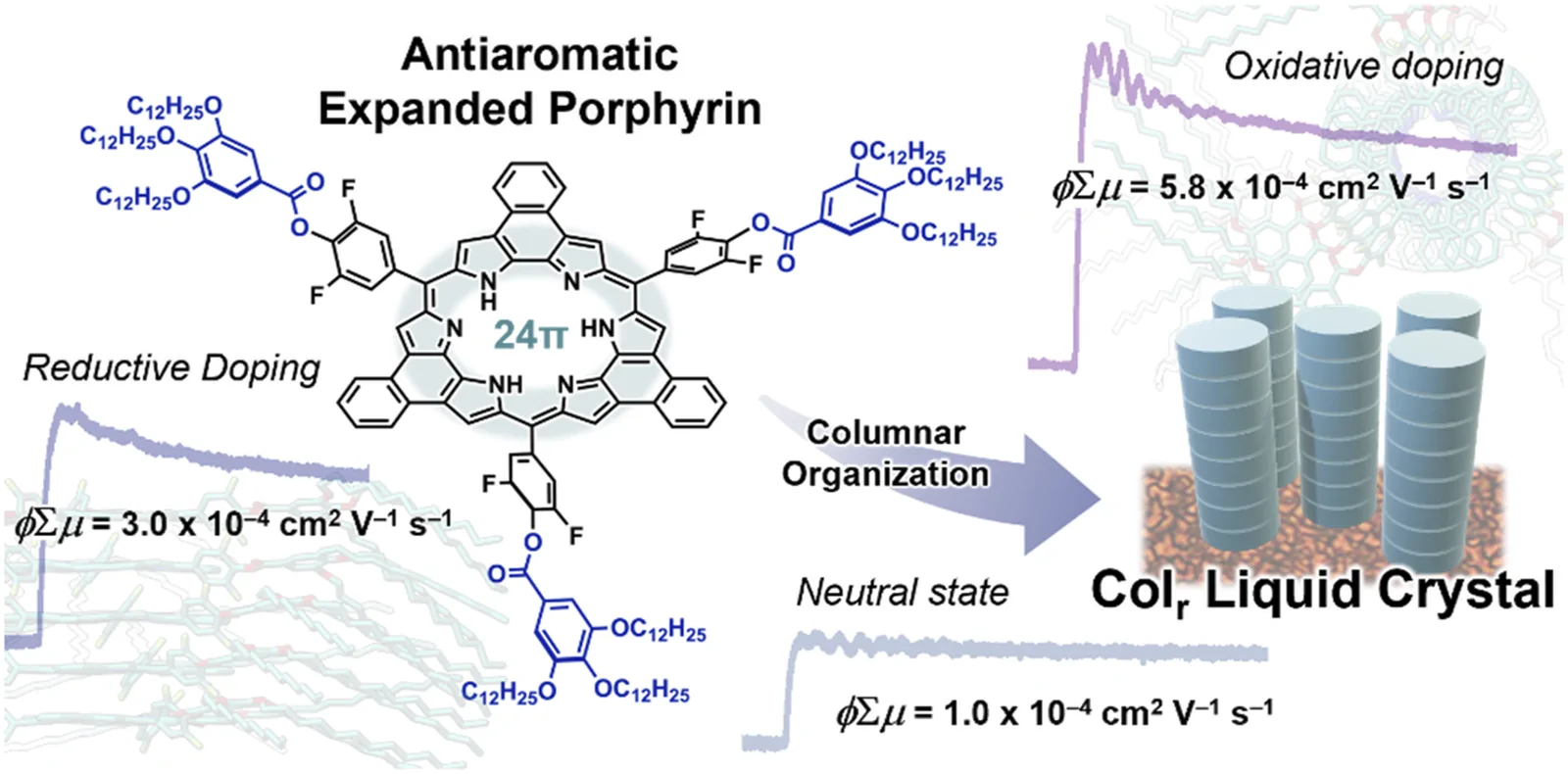

## Introduction

Hierarchical self-assembly of molecules into ordered supramolecular architectures underlies the development of next-generation functional materials.^1,2^ In this context, liquid crystals (LCs) based on π-conjugated molecules are especially attractive, as they combine long-range structural order with intrinsic molecular mobility.^3^ This unique interplay between order and adaptability underpins their potential in electronic and energy-related applications.^4^

The emergent properties of these LCs are largely dictated by their molecular building blocks. Specifically, geometrically π-stacked conjugated cores govern primarily the optoelectronic features, while electronically inactive peripheral aliphatic chains regulate bulk physical characteristics, such as fluidity, phase behavior, and mechanical properties.^5,6^ To date, a wide variety of aromatic systems have been explored as mesogenic cores.^7,8^ In contrast, LC materials based on antiaromatic building blocks remain largely unexplored.^9–11^ Were they routinely available, such systems would be of potential interest due to their distinctive electronic features, including narrow HOMO–LUMO gaps,^12,13^ multi-redox activity,^14^ and potential open-shell character that might support interactions between the individual antiaromatic species.^15,16^ The paucity of LCs based on antiaromatic building blocks reported to date reflects, presumably, (i) the intrinsic instability of most antiaromatic cores, which complicates synthesis and functionalization, (ii) structural distortions driven by a propensity to alleviate antiaromaticity, and (iii) an associated lack of planarity that precludes efficient face-to-face organization.^17,18^

β,β′-Phenylene-bridged hexaphyrins [1.0.1.0.1.0], known as naphthorosarins (NRos) (Fig. 1), have emerged as unique class of expanded porphyrins with potential as mesogenic scaffolds.^19^ NRos possess a 24π-electron antiaromatic core stabilized by an enforced planar geometry imposed by peripheral annulated benzene rings. Moreover, NRos exhibit distinctive redox behavior, reversibly interconverting among 24π-, 25π-, and 26π-electron states *via* proton-coupled electron transfer (PCET) processes upon external stimulation.^20^ This rare combination of antiaromaticity and redox versatility renders NRos attractive as building blocks for LC preparation since the resulting constructs would be expected to display intriguing and tunable optoelectronic features.

**Fig. 1:**
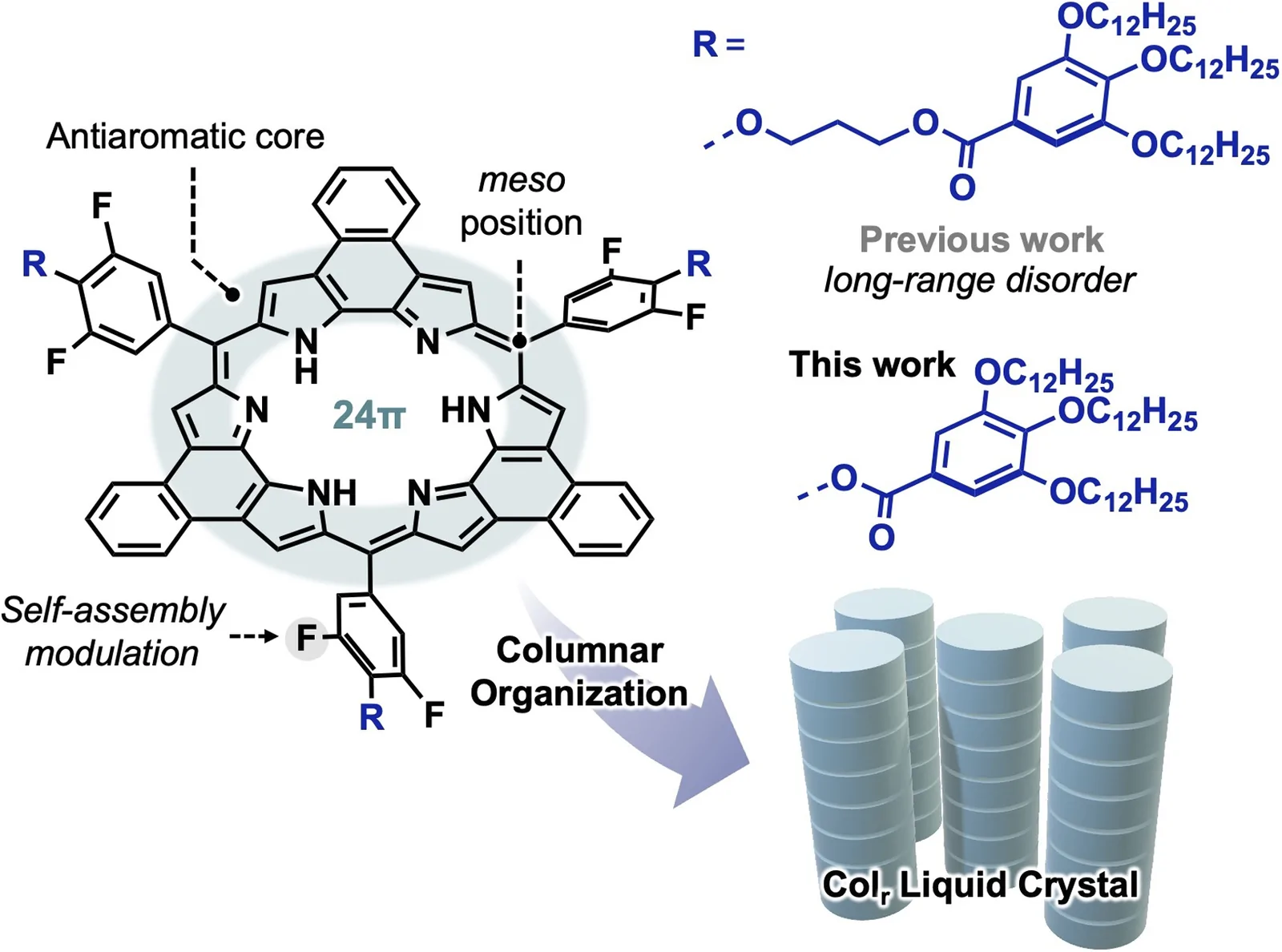
Molecular structure of representative NRos derivatives explored as possible self-assembling units and a 3D model of rectangular columnar phase organization achieved in the present study.

Herein, we report a columnar LC material (NRos1) that is based on a naphthorosarin antiaromatic core. Reflecting, presumably, its redox-active nature, NRos1 displays intrinsic photoconductivity that can be further enhanced *via* either n- or p-doping. These findings unveil a previously unrecognized mode of redox-modulated photoconductivity in LCs based on π-conjugated systems and establish antiaromaticity as a useful design principle in the construction of porphyrinoid-based functional soft materials.

## Results and discussion

### Molecular design, synthesis and characterization

Previous attempts by our group to develop LCs based on naphthorosarins led to the formation of mesophases which, despite exhibiting local cofacial arrangements, did not result in long-range columnar order (Fig. 1).^21^ Given that extended structural organization is essential for efficient charge transport, as well as other practical applications of LCs, we were keen to develop a structurally optimized, scalable, and fully characterized LC system based on NRos. With this goal in mind, we prepared a new naphthorosarin derivative, NRos1, featuring *meso*-aryl substituents bearing long aliphatic chains. Fluorine atoms were introduced into the *meso*-phenyl group to enhance the reactivity of the aldehyde precursor used in the key NRos-forming macrocyclization reaction. The *meta*-fluorinated derivative was chosen over its *ortho*-substituted counterpart to facilitate columnar organization. NRos1 incorporates phenyl gallate bridges between the macrocyclic core and side chains instead of the alkyl gallate linkers used in our original system. This modification was expected to increase the conformational rigidity of the substituents and promote a greater level of mesogenic behavior relative to our first generation system (Fig. 1). As detailed below, these design considerations allowed for the successful preparation of what to our knowledge is the first *bona fide* columnar LC system incorporating an antiaromatic expanded porphyrin core.

As illustrated in Scheme 1, NRos1 was synthesized in good yield from naphthobipyrrole and the aldehyde precursor A1 *via* a one-pot, two-step sequence; this involved: (1) acid-catalyzed macrocyclization; followed by (2) oxidation with DDQ. Notably, the final product was obtained in 15% yield on a ∼200 mg scale, which is comparatively efficient for this class of macrocycles.^22^NRos1 was characterized using ^1^H, ^13^C and ^19^F NMR spectroscopies, as well as mass spectrometry. The ^1^H NMR spectrum of NRos1 in *d*_8_-THF displays features consistent with antiaromaticity, as evidenced by the upfield shift seen for the pyrrolic β-CH signal (5.24 ppm) (Fig. S3.4). Additionally, the UV-vis-NIR absorption spectrum of NRos1 in CH_2_Cl_2_ exhibits broad absorption bands at 400–600 nm and a weak band extending to ∼900 nm (Fig. S5.1). Collectively, these findings are consistent with the antiaromatic character proposed for NRos1.

**Scheme 1:**
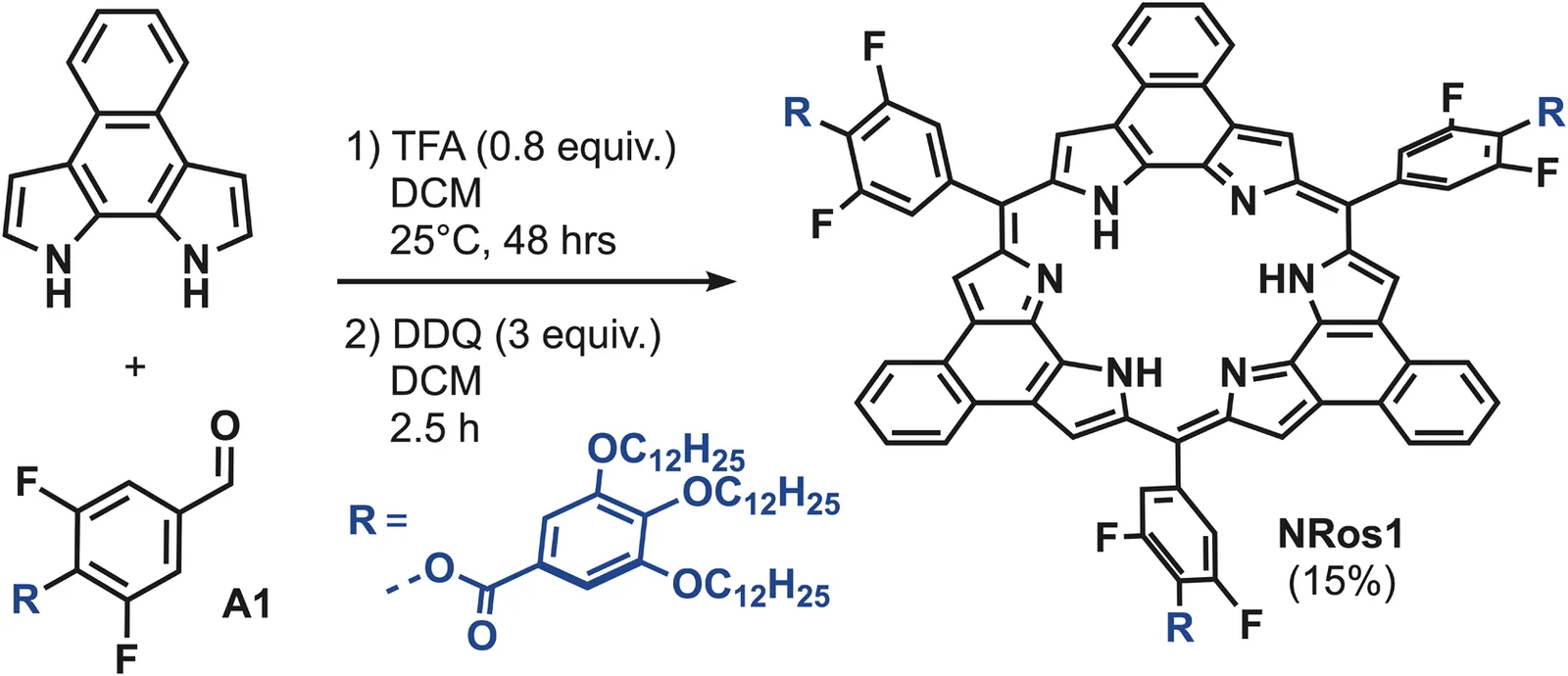
Synthetic route to NRos1.

### Characterization of the columnar liquid crystal

The liquid crystalline behavior of NRos1 was investigated using polarized optical microscopy (POM), differential scanning calorimetry (DSC), and X-ray diffraction (XRD). Initial observation by POM during heating of the as-prepared sample revealed that the material becomes a fully isotropic liquid at approximately 125 °C; additionally, a complex thermogram indicated the presence of several thermal transitions (Fig. S8.1). Entering the mesophase from the isotropic liquid at a cooling rate of 1 °C min^−1^, a texture consistent with a columnar LC phase was observed (Fig. 2a). This state remained stable at room temperature. Despite these POM observations, the cooling thermogram lacked the clearly defined demarcations expected for an isotropic liquid-to-mesophase transition. Thus, NRos1 was subjected to further characterization studies.

**Fig. 2:**
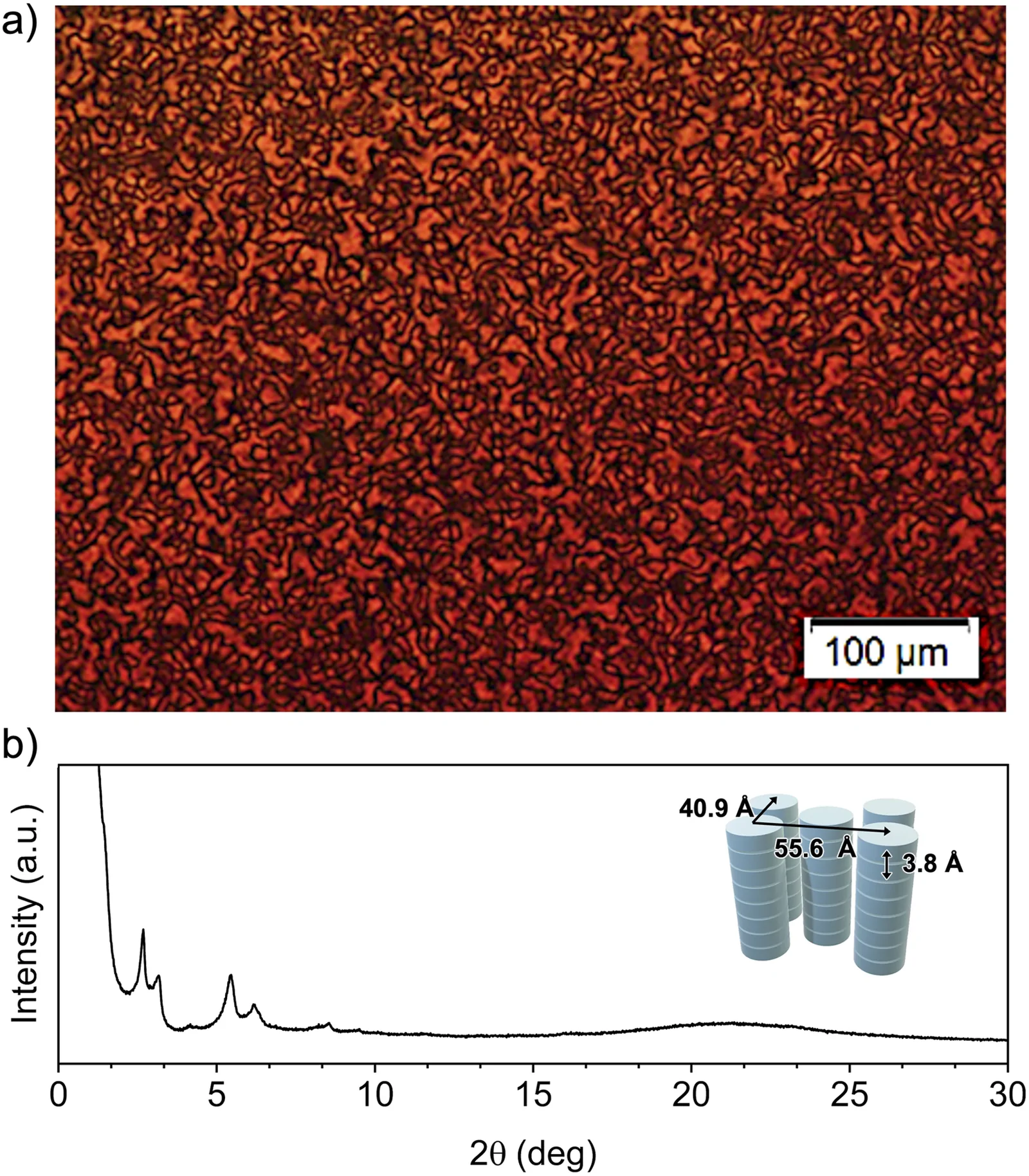
(a) POM microphotograph of NRos1 recorded at 90 °C. (b) XRD diffractogram recorded at room temperature for a cast film after thermal treatment.

The phase transition behavior and the assignment of the columnar phase as a LC were made on the basis of XRD analyses. A diffraction pattern was recorded for a thin film prepared by drop-casting, followed by thermal treatment consisting of heating to 125 °C and subsequent cooling to room temperature at a rate of 5 °C min^−1^ (Fig. 2b). This pattern was characterized by a broad diffuse halo at wide angles. This feature was taken as evidence for the formation of an LC phase. The broad wide-angle reflection corresponding to a *d*-spacing centered at 3.8 Å was assigned to the average π–π stacking distance between adjacent molecules. In the small-angle region, a series of periodic reflections (see Table S1) could be readily indexed to a rectangular columnar (Col_r_) mesophase, yielding lattice parameters of *a* = 40.9 Å and *b* = 55.6 Å. These XRD data are consistent with the formation of a rectangular columnar (Col_r_) LC phase that contains one molecule per stacking unit within the column.

Supramolecular pentamers of NRos1 were optimized at the GFN2-xTB level^23^ in the gas phase to model the preferred packing and assess the extent to which the present design would favor columnar self-assembly (Fig. 3). The optimized pentamer revealed a highly symmetric, co-axial and helical columnar assembly (Fig. 3a). The interplanar distance between adjacent NRos1 cores was calculated to be 3.40 Å (Fig. 3b). This value is consistent with what was previously observed in single-crystal XRD studies of crystalline NRos samples.^24^ However, it is slightly smaller than the average stacking distance inferred from the XRD studies discussed above (*ca.* 3.8 Å). Presumably this difference reflects the molten-like, conformationally mobile periphery of NRos1 and associated thermal disorder that would be expected to increase the mean core–core separation. The computational studies further revealed that the NRos1 building blocks retain their intrinsic planarity within the supramolecular column. This behavior stands in contrast with what was seen in the case of our previously reported LCs, wherein macrocycle distortions were required to enable a cofacial arrangement of the subunits.^21^ The preservation of coplanarity in NRos1 highlights the effectiveness of the present molecular design strategy and its utility in supporting the formation of a Col_r_ phase.

**Fig. 3:**
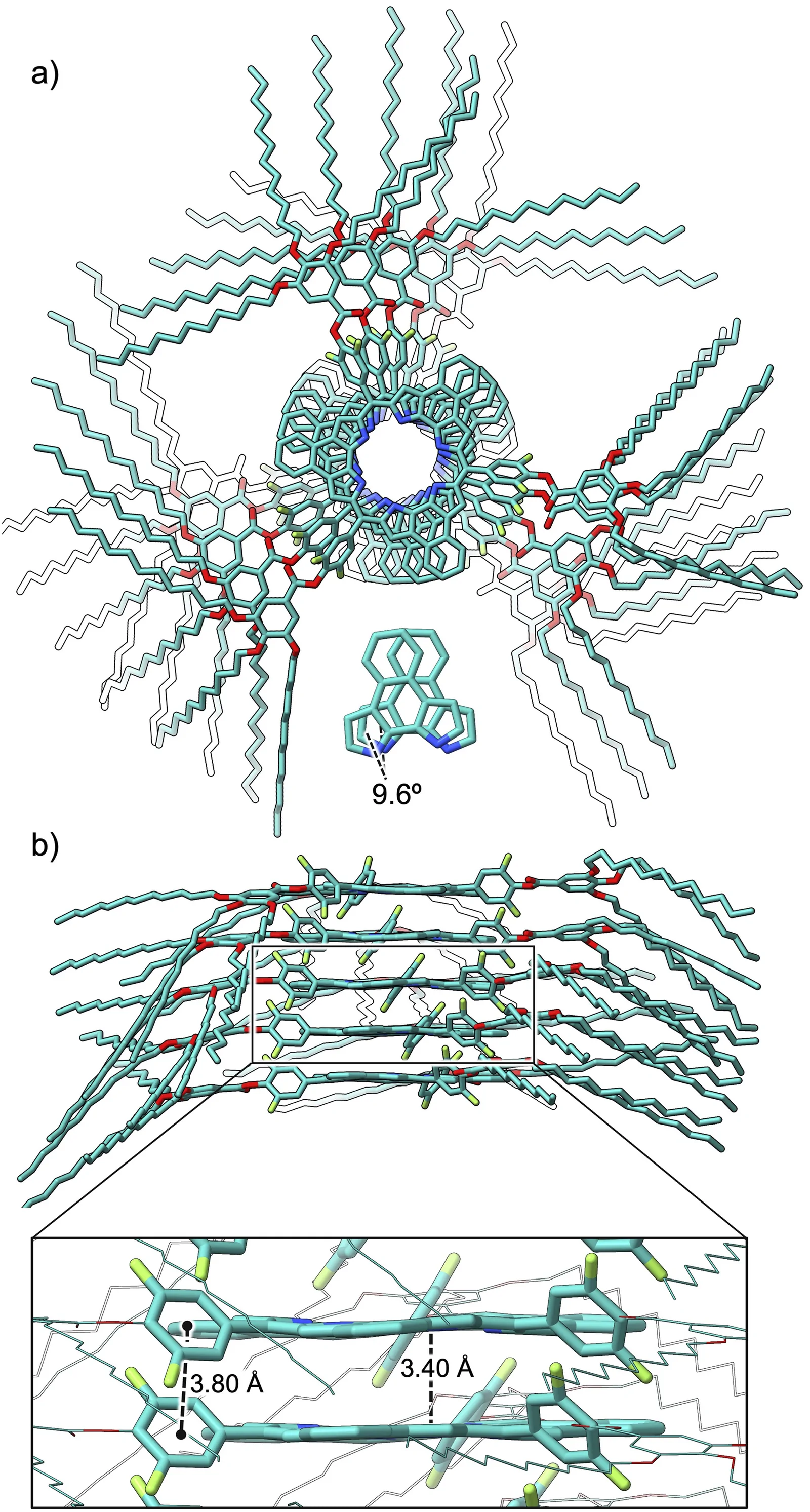
(a and b) Top and side view of the GFN2-xTB-optimized geometry calculated for a supramolecular pentamer of NRos1 showing calculated twist angle and interplanar distance.

A small rotational offset of 9.6° between adjacent NRos1 cores along the stacking axis was observed. This value is significantly smaller than the ∼30° twist commonly found in related systems.^25^ Furthermore, the peripheral chains cluster in close proximity, likely facilitated by the rigidity of the phenyl gallate units. We hypothesize that this chain clustering may be the primary driving force directing the columnar assembly of NRos1. In particular, the solvophobic-like segregation may favor a cofacial arrangement of the antiaromatic cores over alternative geometries (*e.g.*, T-shaped or parallel-displaced). Within this framework, the precise interplanar spacing and rotational offset would then reflect a secondary balance between dispersion, favored by the large planar π-surface of the macrocycle, and Pauli exchange-repulsion, which limits the minimum approach distance between adjacent cores, rather than reflecting any distinct π-orbital-based attractive force.^25^ This synergistic effect of short interplanar spacing, minimal rotational offset, and organized side chain packing aligns well with the well-defined columnar organization observed experimentally.

### Charge transport properties and photoconductivity modulation

Prompted by the columnar organization of NRos1, we next investigated its charge-transport properties. This was done using flash-photolysis time-resolved microwave conductivity (FP-TRMC). This technique provides a direct measure of intrinsic photoconductivity, expressed as *ϕ*Σ*µ*, where *ϕ* denotes the charge-carrier generation quantum yield and Σ*µ* represents the sum of hole and electron mobilities (Σ*µ* = *µ*_h_ + *µ*_e_).^26^ FP-TRMC is particularly attractive in the case of LCs since it enables monitoring of photoconductivity after specific thermal treatments. It thus allows direct comparisons between condensed phases with different degrees of structural order at the nanoscale.

In the present study, films of NRos1 were examined both in their as-prepared amorphous state and after thermal treatment to induce columnar organization (*i.e.*, heating to the isotropic phase at *ca.* 125 °C followed by cooling to room temperature). FP-TRMC measurements were performed upon excitation at 355 nm under aerobic conditions at room temperature. The transient conductivity trace of the amorphous state exhibited a low *ϕ*Σ*µ*_max_ value of 0.1 × 10^−4^ cm^2^ V^−1^ s^−1^, whereas the thermally treated sample showed a tenfold enhancement, reaching 1.0 × 10^−4^ cm^2^ V^−1^ s^−1^ (Fig. 4). This latter value is similar to those reported for other porphyrinoid-based columnar materials, such as supramolecular polymers based on Ni(ii) norcorroles (antiaromatic) or Zn(ii) porphyrin (aromatic), which exhibit *ϕ*Σ*µ*_max_ values of 0.5–1.0 × 10^−4^ cm^2^ V^−1^ s^−1^ under similar conditions.^27^ These results suggest that the quasi face-centered stacking of the antiaromatic subunits within the columnar array provides an efficient charge transport pathway. Notably, the photogenerated charge carriers display relatively long lifetimes of 23 µs. We rationalize this lifetime in terms of relatively inefficient charge recombination, as well as poor electron transfer between the individual columns, which are separated by high dielectric domains resulting from the peripheral substituents.

**Fig. 4:**
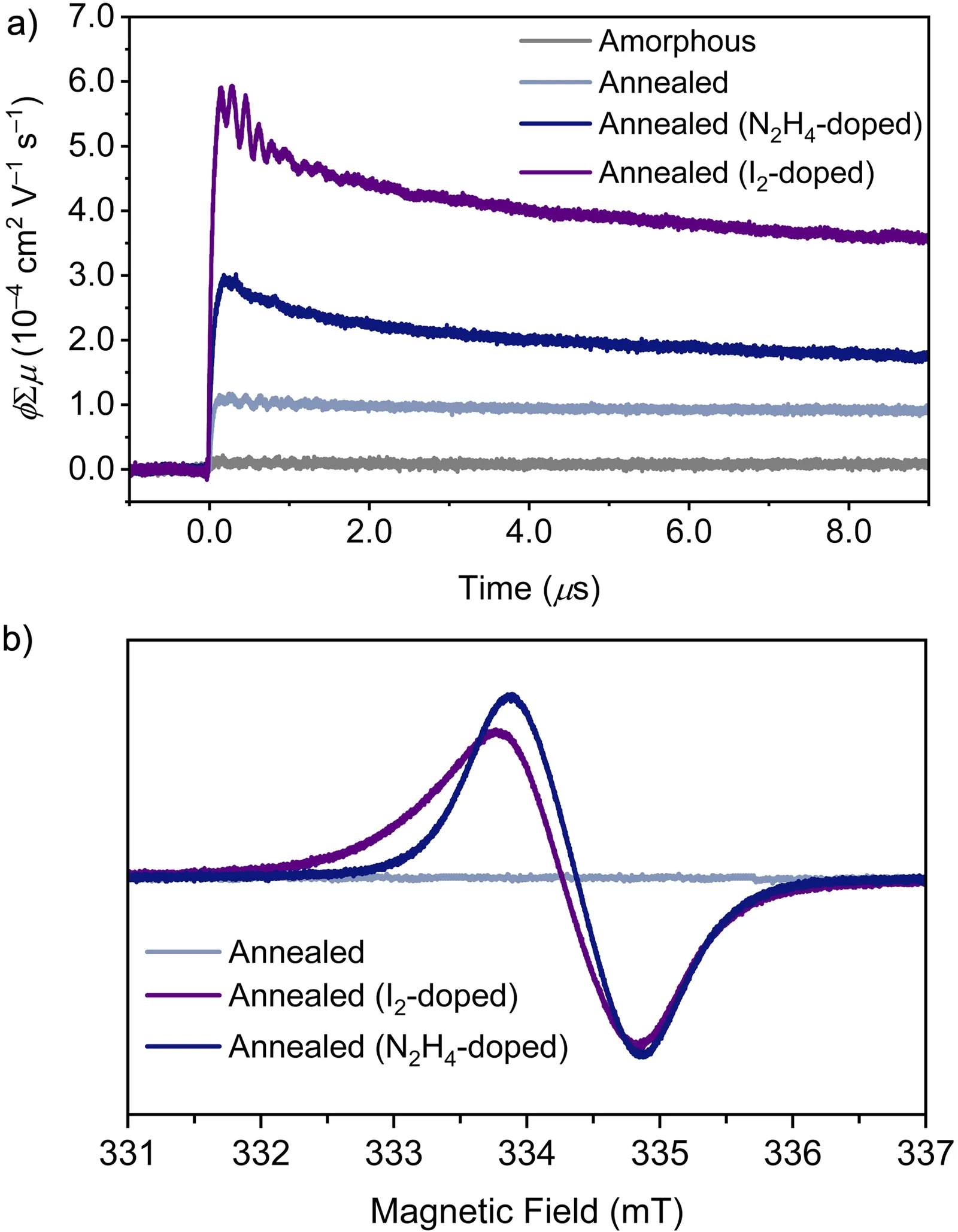
(a) FP-TRMC transient photoconductivity traces of NRos1 films in the as-prepared amorphous state (gray) and after thermal treatment without (light blue) and with N_2_H_4_ (navy) and I_2_ (purple) doping. Measurements were performed on quartz substrates upon excitation at 355 nm (9.1 × 10^15^ photons cm^−2^). Charge-carrier lifetimes of 23, 25, and 23 µs were obtained for the thermally treated films by fitting a single-exponential decay to the maximum *ϕ*Σ*µ*. (b) Corresponding EPR spectra of annealed NRos1 films before and after doping.

Guided by cyclic voltammetry (CV) studies, which showed the first reduction and first oxidation of NRos1 in CH_2_Cl_2_ solution at *E*_1/2_ = −0.76 and 0.15 V *vs.* Fc/Fc^+^, respectively (Fig. S6.1), we envisaged that the photoconductivity of NRos1 could be modulated through both p- and n-type chemical doping. Following procedures previously reported by our group and others, for n-doping, thermally treated films were exposed (30 min) to N_2_H_4_ vapor (E(N_2_/N_2_H_4_) = −0.73 V *versus* Fc/Fc^+^).^28^ p-Doping was achieved by exposure (30 min) to an I_2_ atmosphere (*E*(I_2_/I^−^) = 0.15 V *versus* Fc/Fc^+^).^29^ As shown in Fig. 4a, both n- and p-doping led to an enhancement in the photoconductivity, with approximately threefold (3.0 × 10^−4^ cm^2^ V^−1^ s^−1^) and nearly sixfold (5.8 × 10^−4^ cm^2^ V^−1^ s^−1^) increases being observed, respectively. In both cases, the charge-carrier lifetimes remained essentially unchanged (25 and 23 µs, respectively).

It is worth noting that the relatively high *ϕ*Σ*µ*_max_ values observed here are comparable to, and in some cases even surpass those reported for systems with a higher degree of structural order and rigidity. For instance, the photoconductivity values were reported on the order of 10^−5^–10^−4^ cm^2^ V^−1^ s^−1^ in conjugated two-dimensional covalent organic frameworks (2D COFs),^30,31^ metal–organic frameworks (MOFs),^32,33^ and crystalline lattices^34,35^ under almost identical molecular doping conditions. These results lead us to suggest that tailored NRos macrocycles could emerge as promising building blocks for the construction of novel semiconducting materials or in future device-related applications.

To gain insights into the doping process, electron paramagnetic resonance (EPR) spectroscopic studies were performed using thin films of NRos1 exposed to either I_2_ (p-doping) or N_2_H_4_ (n-doping) at room temperature. In both cases, the emergence of intense, isotropic EPR signals was observed. These features were centered at *g* = 2.0061 (p-doping) and *g* = 2.0053 (n-doping) (Fig. 4b). No hyperfine structure could be resolved in either spectrum, supporting the formation of highly delocalized paramagnetic species. The EPR spectral signatures of the doped species are thus consistent with radical states extending over the π-conjugated framework and closely resemble those previously reported for proton-coupled electron transfer (PCET) processes in NRos derivatives in solution.^19^ The small difference in *g*-value for the doped samples likely arises from the distinct π-conjugation of the two radicals—a 23π-electron species upon p-doping *vs.* a 25π-electron species upon n-doping. Based on these results, we conclude that both oxidative and reductive treatments generate stabilized, open-shell radical species within the columnar assemblies.

Further insight into the doping process came from solid-state UV-vis-NIR absorption spectroscopy. The undoped films display a broad absorption band spanning the 400–600 nm spectral region, with a maximum at 475 nm (Fig. S5.2). This feature is broadened compared to the solution spectrum, which may reflect a combination of structural heterogeneity inherent to the columnar mesophase and excitonic coupling between neighboring NRos1 cores within the columnar assembly. Upon exposure to N_2_H_4_, a pronounced bathochromic shift is observed, giving rise to a new absorption feature extending from 500 to 800 nm. By comparison with the spectral evolution of monomeric analogues, we assign these changes to the formation of a reduced 25π-electron species *via* a PCET mechanism.^19^ In contrast, exposure to I_2_ induces more subtle, yet clearly discernible, spectral changes (Fig. S5.3), including a *ca.* 100 nm redshift of the absorption maximum and the appearance of a broad, low-intensity band in the near-infrared region (*ca.* 1100 nm).

As a complement to the above studies, potential-dependent thin-layer UV-vis-NIR spectroelectrochemistry was conducted on a reference system (NRosF_5_) in dichloromethane containing 0.1 M TBAPF_6_. As shown in Fig. S6.2, this oxidation results in a modest perturbation of the main absorption band (*ca.* 20 nm redshift in the absorption maximum) that is accompanied by the emergence of a weak, broad feature centered at 836 nm. Although the magnitude of the bathochromic shift is more pronounced in the solid state-a phenomenon likely arising from enhanced excitonic coupling in the columnar assembly^36^ – the overall spectral evolution is consistent with that observed under conditions of controlled electrochemical oxidation in solution. Importantly, these changes cannot be ascribed to dopant-derived species, since the I_3_^−^/I_5_^−^ polyiodides absorb only up to *ca.* 400 nm and in the visible region the doped film shows a decrease in absorbance and an absence of new features in this portion of the spectrum. The N_2_H_4_ used for n-doping is, in turn, transparent above *ca.* 250 nm. Taken together with the EPR data, these results lead us to conclude that both p- and n-doping result in the generation of radical species whose formation underpins the observed enhancements in photoconductivity.

To assess whether the enhanced photoconductivity originates from changes in the core-to-core distance, supramolecular dimers of p- and n-doped NRos1 (*i.e.*, 23π- and 25π-electron species, respectively) were optimized at the GFN2-xTB level. As shown in Fig. S7.1, the interplanar distances between adjacent NRos1 cores remain similar to that of the neutral dimer in both cases, ruling out a geometrically induced enhancement of *ϕ*Σ*µ*. On this basis, we propose that the enhanced photoconductivity primarily arises from the electronic effects of doping, whereby partial depopulation or population of the frontier-orbital manifold facilitates charge transport, as commonly proposed for doped organic semiconductors.^37^

## Conclusions

In summary, we report here a columnar LC based on a readily accessible, planar antiaromatic expanded porphyrinoid (NRos1). As confirmed by FP-TRMC measurements, the columnar organization of NRos1 enables efficient charge-carrier generation and transport upon 355 nm irradiation, affording relatively high *ϕ*Σ*µ*_max_ values of up to 1.0 × 10^−4^ cm^2^ V^−1^ s^−1^ as compared to the amorphous state (0.1 × 10^−4^ cm^2^ V^−1^ s^−1^). These values are comparable to those reported for norcorrole- and porphyrin-based columnar arrays. Together with the experimentally observed core-to-core distances, this similarity leads us to suggest that no additional stabilization arises from specific interactions between the antiaromatic π-surfaces and that the charge-transport properties of NRos1 primarily originate from coaxial stacking of the π-conjugated cores. Nevertheless, capitalizing on the redox versatility inherent in the NRos core, this photoconductivity could be further enhanced through both n- and p-doping, reaching values as high as 5.8 × 10^−4^ cm^2^ V^−1^ s^−1^. The present study thus positions antiaromatic expanded porphyrinoids as promising platforms for the creation of new optoelectronic soft materials. Ongoing efforts aim to harness the antiaromaticity of NRos and other porphyrin analogues for on-device applications and to create LCs with high-value electronic features.

## Author contributions

D. D. N.: investigation, writing – original draft; W. R. O.: investigation, writing – review & editing; A. C.: investigation, writing – review & editing; T. S.: conceptualization, funding acquisition, writing – review & editing; J. L.: conceptualization, investigation, supervision, writing – original draft; S. S.: conceptualization, funding acquisition, writing – review & editing; J. L. S.: conceptualization, funding acquisition, writing – review & editing.

## Conflicts of interest

There are no conflicts to declare.

## Supplementary Material

SC-017-D6SC06405A-s001

## Data Availability

The data supporting this article have been included as part of the supplementary information (SI). Supplementary information is available. See DOI: https://doi.org/10.1039/d6sc06405a.
