# Supplementary material for: Antiaromatic expanded porphyrin enables redox-tunable photoconductivity in columnar liquid crystals
Source: Chem Sci. 2026 Sep 14;17(43):20945–50. doi: 10.1039/d6sc06405a (PMC13737591; doi:10.1039/d6sc06405a)
Supplement: Supplementary file 1 [file SC-017-D6SC06405A-s001.pdf]

Supplementary Information (SI) for Chemical Science.
This journal is © The Royal Society of Chemistry 2026

Supplementary Information

Antiaromatic Expanded Porphyrin Enables Redox-Tunable Photoconductivity in Columnar Liquid Crystals

Duong D. Nguyen,[a] W. Ryan Osterloh,[b] Alberto Concellón,[c] Teresa Sierra,*[c] Jorge Labella*[b], Shu Seki,*[b] and Jonathan L. Sessler*[a]

[a] Duong D. Nguyen, Prof. Dr. Jonathan L. Sessler
Department of Chemistry, The University of Texas at Austin, 105 East 24th Street, Austin, Texas 78712-1224, USA
E-mail: sessler@cm.utexas.edu

[b] Dr. W. Ryan Osterloh, Dr. Jorge Labella. Prof. Dr. Shu Seki
Department of Chemical Science and Engineering, Graduate School of Engineering, Kyoto University, Nishikyo-ku, Kyoto 615-8510, Japan.
E-mail: labella.jorge.54u@st.kyoto-u.ac.jp
seki@moleng.kyoto-u.ac.jp

[c] Dr. Alberto Concellón, Dr. Teresa Sierra
Instituto de Nanociencia y Materiales de Aragón (INMA), Departamento de Química Orgánica, Facultad de Ciencias, CSIC-Universidad de Zaragoza, Zaragoza 50009, Spain
E-mail: tsierra@unizar.es

Table of Contents

1. Instrumentation and Materials 2
2. Synthetic Procedures and Characterization Data 3
   Synthesis and Characterization of **A1** 3
   Synthesis and Characterization of Naphthorosarin **NRos1** 4
3. NMR Spectra 5
4. Mass Spectra 7
5. UV-Vis Spectra 8
6. Electrochemistry and Spectroelectrochemistry 9
7. Computational Studies 10
8. Differential Scanning Calorimetry 11
9. X-ray Diffraction Studies 12
10. Flash-Photolysis Time-Resolved Microwave Conductivity (FP-TRMC) 12
11. Supporting References 13

1. Instrumentation and Materials

Reaction progress was monitored by thin layer chromatography (TLC), employing aluminum sheets coated with silica gel type 60 F254 (0.2 mm thick, Merck) visualized using either a 254 or 365 nm UV lamp. Purification and separation of the synthesized products was performed by normal-phase column chromatography, using silica gel (230–400 mesh, 0.040–0.063 mm, Merck) as the solid support and size exclusion chromatography, using Bio-Beads^{TM} S-X1 (styrene divinylbenzene beads, 40-80 μm bead size, Bio-Rad). Eluents, along with the relative ratios in the case of solvent mixtures, are indicated for each particular case. Nuclear magnetic resonance spectra (^{1}H, ^{13}C, ^{19}F NMR spectra) were recorded on Agilent MR400, Bruker AV-400, Bruker DRX-500 or Varian MR600 spectrometers at the Department of Chemistry of The University of Texas at Austin (UT Austin). The deuterated solvent employed in each case is indicated in brackets, and its residual peak was used to calibrate the spectra using literature reference δ ppm values. The peaks marked with asterisks indicates residual solvent signals. The NMR spectroscopic solvents were purchased from Cambridge Isotope Laboratories or Fischer Scientific. All spectra were recorded at room temperature. High resolution ESI mass spectrometry was carried out using an Ion Spec Fourier Transform mass spectrometer (9.4 T). Electrospray ionization (ESI) mass spectra were recorded on an Thermo Fischer Scientific APCI-orbitrap-MC Exactive Plus instrument housed in the Department of Molecular Engineering, Kyoto University. Atmospheric Pressure Chemical Ionization (APCI) mass spectra were recorded on an Agilent Technologies 6530 Accurate-Mass Q-TOF instrument housed in the Department of Chemistry, UT Austin. Ultraviolet-visible (UV-Vis) spectra were recorded using spectroscopic grade solvents using a Varian Cary 5000 spectrophotometer housed in the in the Department of Chemistry, UT Austin. The logarithm of the molar extinction coefficient (ε) is indicated in brackets for each maximum. Cyclic voltammetry (CV) and differential pulse voltammetry (DPV) were carried out at 298K using a BAS ALS600D electrochemical analyzer. Liquid crystal behavior was investigated by polarized-light optical microscopy (POM) using an Olympus BH-2 polarizing microscope fitted with a Linkam THMS600 hot stage. Thermogravimetric analysis (TGA) was performed using a TA Q5000IR instrument at a heating rate of 10 ºC /min under a nitrogen atmosphere. The temperatures and enthalpies of the phase transitions were determined by calorimetric measurements performed with a differential scanning calorimeter (DSC) TA Instrument Q2000 system. X-ray diffraction (XRD) measurements were performed using a PANalytical Empyrean diffractometer (CuKα1 = 1.5409 Å) equipped with a Reflection-Transmission spinner stage. X-band EPR spectra were recorded at room temperature under ambient conditions using a JEOL JES-FA200 instrument. Spectra were taken before and after doping the as-prepared LC which was accomplished via exposure to either excess I_2 or N_2H_4 vapor for 30 minutes. The microwave frequency (9.445 GHz), microwave power (1 mW), and magnetic-field modulation amplitude (0.1 mT) were kept constant for each experiment. The respective g-values were determined by two-point calibration using the third and fourth hyperfine lines of Mn^{2+} (I = 5/2) supplied as an internal reference (Mn^{2+}/MgO, JES-FA200).

Dry solvents were purchased from commercial suppliers as anhydrous-grade or thoroughly dried before using standard methods. Solid, hygroscopic reagents were dried in a vacuum oven before use. Chemicals were purchased from commercial suppliers and used without further purification. Dichloromethane (CH_2Cl_2, anhydrous, 99.5%) for electrochemistry was purchased from Wako Chemicals and used as received. Tetrabutylammonium hexafluorophosphate ($TBAPF_6$, 98%) was purchased from Tokyo Chemical Industry and recrystallized from hot EtOH followed by drying *in vacuo* at 60 °C prior to use.

Thin-film samples were prepared by drop-casting, followed by thermal treatment consisting of heating to 125 ºC followed by subsequent cooling to room temperature at a rate of 5 ºC/min.

2. Synthetic Procedures and Characterization Data

The synthesis and characterization of naphthobipyrrole and **NRosF$_5$** has been previously reported.[1,2]

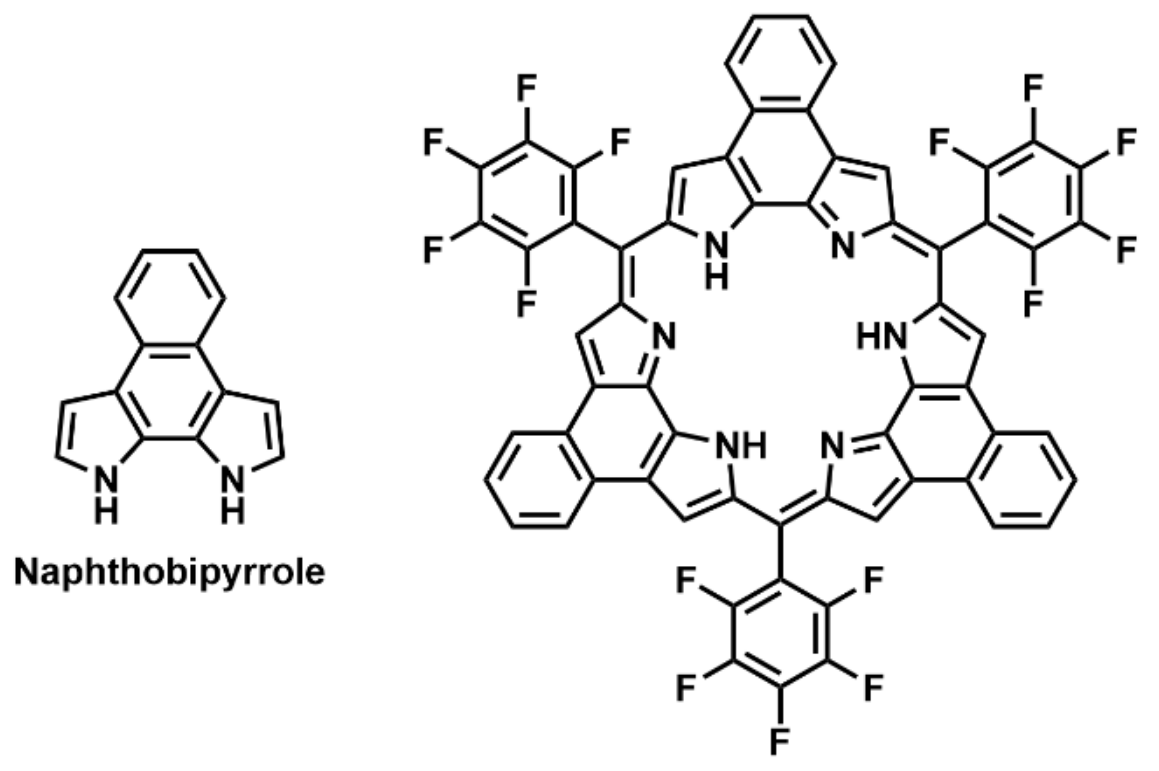

NRosF$_5$

Synthesis and Characterization of **A1**

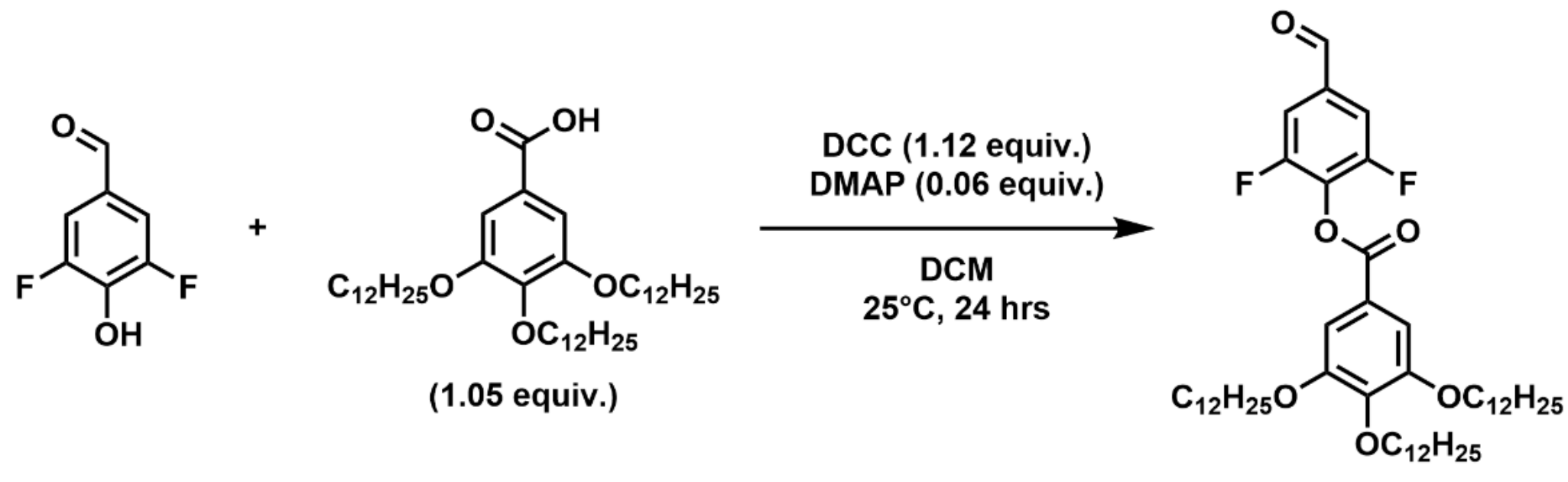

A1

In a 250 mL round bottom flask, a mixture of 3,5-difluoro-4-hydroxybenzaldehyde (1 g, 6.32 mmol, 1 equiv.), 4-dimethylaminopyridine (DMAP, 46.4 mg, 0.38 mmol, 0.06 equiv.) and 3,4,5-tris(dodecyloxy)benzoic acid (4.48 g, 6.64 mmol, 1.05 equiv.) was dissolved in a mixture of dichloromethane (DCM; 60 mL). The solution was then deoxygenated for 10 minutes by bubbling with nitrogen gas. To the resulting solution, a solution of 1,3-dicyclohexylcarbodiimide (DCC, 1.46 g, 7.1 mmol, 1.12 equiv.) in DCM (20 mL) was added dropwise. The resulting mixture was then stirred at 25°C for 24 hours. Once the reaction was deemed complete based on a TLC analysis, the reaction mixture was concentrated to remove the volatiles. The residue was diluted with water (50 mL) and extracted with DCM (20 mL x3). The combined organic fractions were then washed with brine (20 mL) and water (20 mL) before being dried over Na_2SO_4 and concentrated *in vacuo*. The resulting crude product was purified via silica gel column chromatography using hexanes: ethyl acetate = 10:1 as the eluent.

Yield 4.12 g, 80%, white solid.

^{1}H NMR (400 MHz, $CDCl_3$) δ 9.94 (s, 1H), 7.58 (d, *J* = 6.9 Hz, 2H), 7.41 (s, 2H), 4.06 (dt, *J* = 11.1, 6.6 Hz, 6H), 1.87 – 1.72 (m, 6H), 1.48 (t, *J* = 7.9 Hz, 6H), 1.27 (dd, *J* = 9.9, 5.1 Hz, 48H), 0.88 (td, *J* = 6.9, 2.0 Hz, 9H).

^{13}C NMR (100 MHz, $CDCl_3$) δ 188.74, 162.63, 157.27, 154.73, 153.18, 143.90, 134.45, 132.91, 121.59, 113.24, 113.01, 109.09, 73.76, 69.38, 32.03, 32.01, 30.43, 29.82, 29.78, 29.75, 29.71, 29.65, 29.46, 29.34, 26.16, 26.13, 22.78, 14.20.

^{19}F NMR (376 MHz, $CDCl_3$) δ -122.06 (d, J = 6.9 Hz).

HRMS (ESI positive): *m/z* [M+H$^+$] Calculated for $C_{50}H_{80}F_2O_6$: 815.5996, found: 815.6004.

Synthesis and Characterization of Naphthorosarin **NRos1**

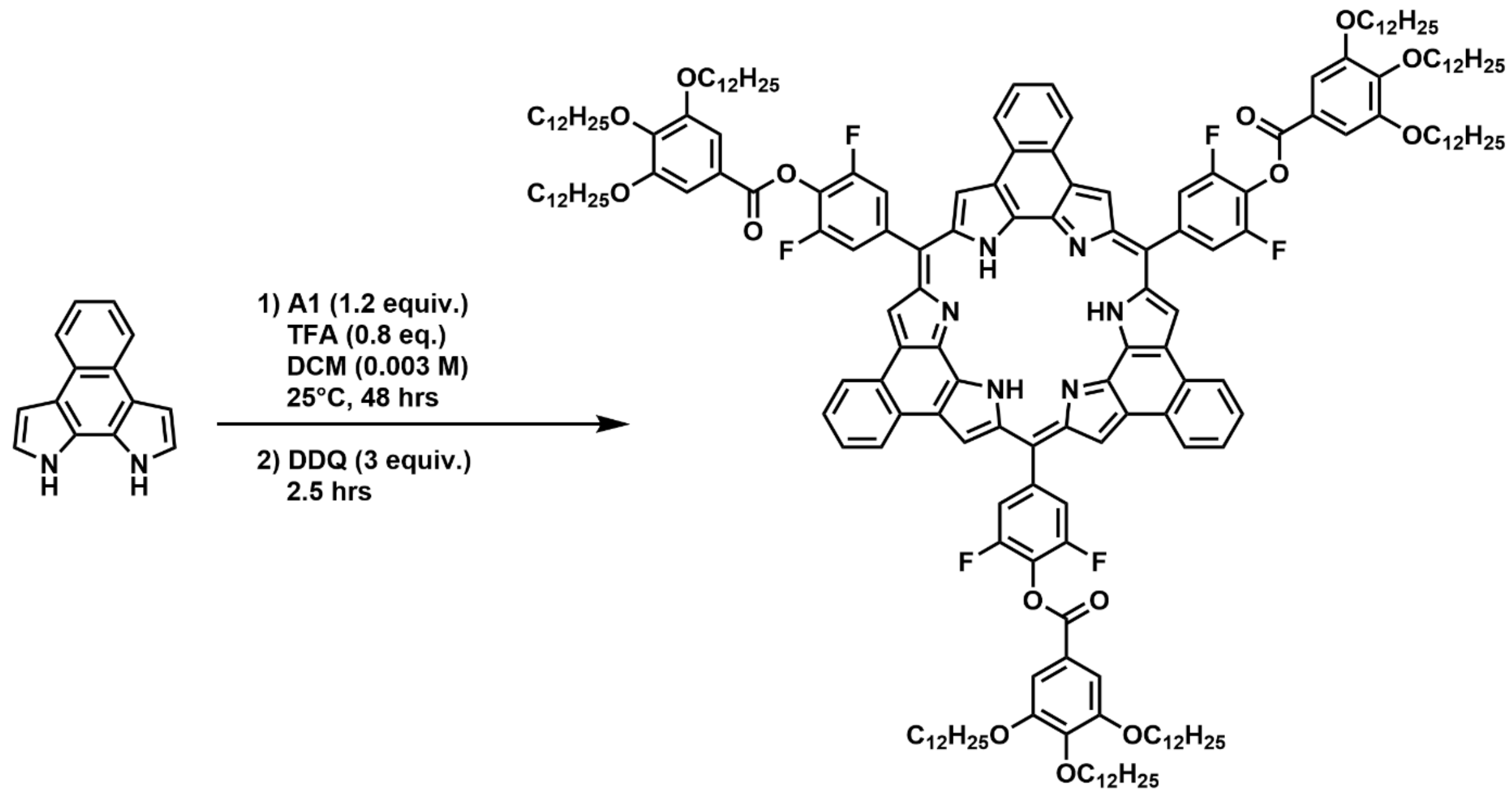

A 1 L round-bottomed flask was charged with naphthobipyrrole (300 mg, 1.45 mmol, 1 equiv.) and **A1** (1.42 g, 1.75 mmol, 1.2 equiv.). Dry DCM (900 mL, 0.0015 M) was then added to dissolve the solids. The reaction mixture was bubbled with nitrogen for 15 minutes and the flask fully covered with aluminum foil. Trifluoroacetic acid (90 µL, 1.17 mmol, 0.8 equiv.) was then slowly added via syringe. The reaction mixture was stirred at room temperature for 48 hours under a nitrogen atmosphere. After this time, DDQ (1056 mg, 4.65 mmol, 3.2 equiv.) was added, and the reaction mixture was stirred for a further 2.5 hours open to air and light. Once the reaction was deemed complete based on a TLC analysis, excess triethylamine (1.5 mL) was added to the reaction flask and the whole solution was passed through a plug of neutral alumina. Excess DCM (about 500 mL) was used to elute fully the dark purple fraction. The resulting solution was then evaporated to dryness. The crude product obtained in this way was purified via silica gel column chromatography (doped with 1% triethylamine) using hexanes: dichloromethane = 1:1 to 1:3 gradient as the eluent. The product obtained in this way was then further purified via size exclusion chromatography (Bio-Beads^{TM} S-X1) using DCM as the eluent. The product obtained in this way was subjected to recrystallization from DCM/methanol-water to give the final product.

Yield 219 mg, 15%, dark brown solid.

^{1}H NMR (600 MHz, THF-d_8) δ 7.40 (s, 6H), 7.11 (dd, J = 6.0, 3.4 Hz, 6H), 6.98 (dt, J = 6.0, 3.7 Hz, 6H), 6.86 (d, J = 8.7 Hz, 6H), 5.24 (s, 6H), 4.12 (dt, J = 13.6, 6.6 Hz, 18H), 1.92 (t, J = 7.3 Hz, 9H), 1.79 (p, J = 6.5 Hz, 9H), 1.57 (p, J = 7.2 Hz, 18H), 1.42 – 1.28 (m, 144H), 0.90 – 0.87 (m, 27H).

^{13}C NMR (151 MHz, THF-d_8) δ 163.92, 158.02, 156.36, 156.33, 155.08, 151.75, 150.93, 145.85, 143.78, 136.02, 135.96, 133.00, 129.72, 129.62, 129.51, 127.74, 125.80, 125.13, 123.53, 122.55, 114.38, 114.24, 110.66, 110.44, 74.87, 70.86, 33.75, 33.73, 32.29, 31.63, 31.58, 31.52, 31.48, 31.44, 31.28, 31.20, 31.17, 28.00, 27.98, 24.42, 24.41, 15.29.

^{19}F NMR (564 MHz, THF-d_8) δ -124.20.

HRMS (FT-APCI-MS positive): *m/z* [M+H$^+$] Calculated for $C_{192}H_{258}F_6N_6O_{15}$: 3002.9587, found: 3002.9696.

3. NMR Spectra

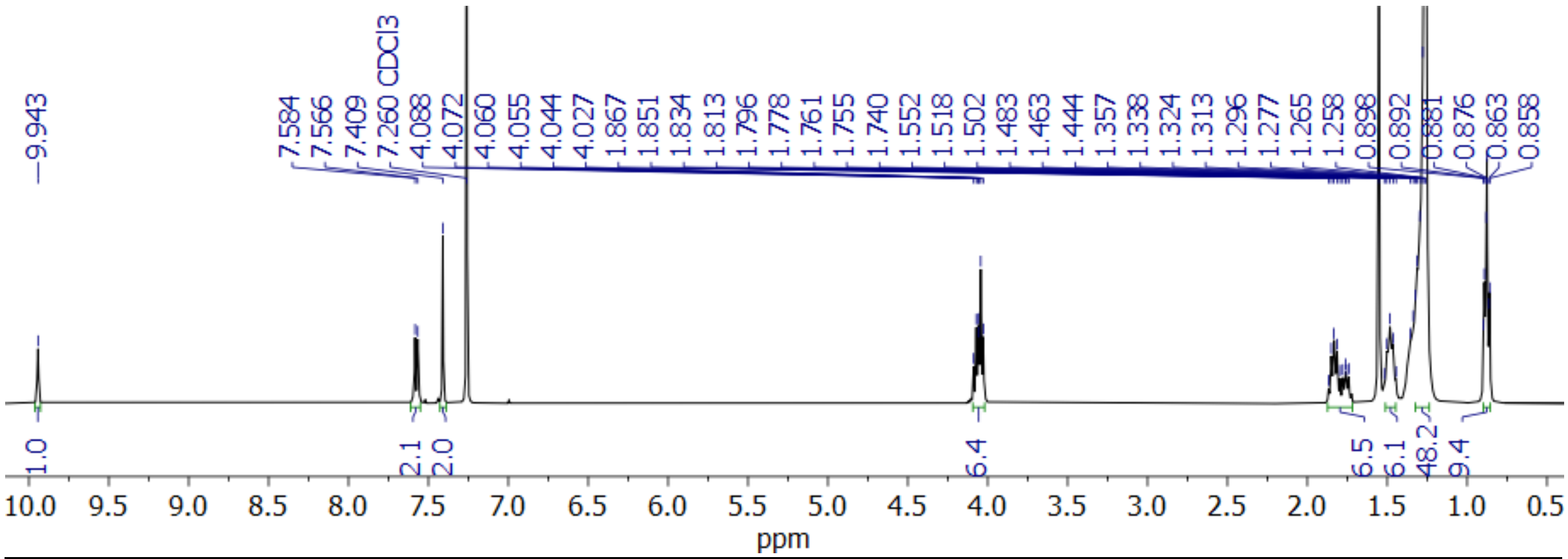

Figure S3.1. ^{1}H NMR spectrum ($CDCl_3$) of **A1**.

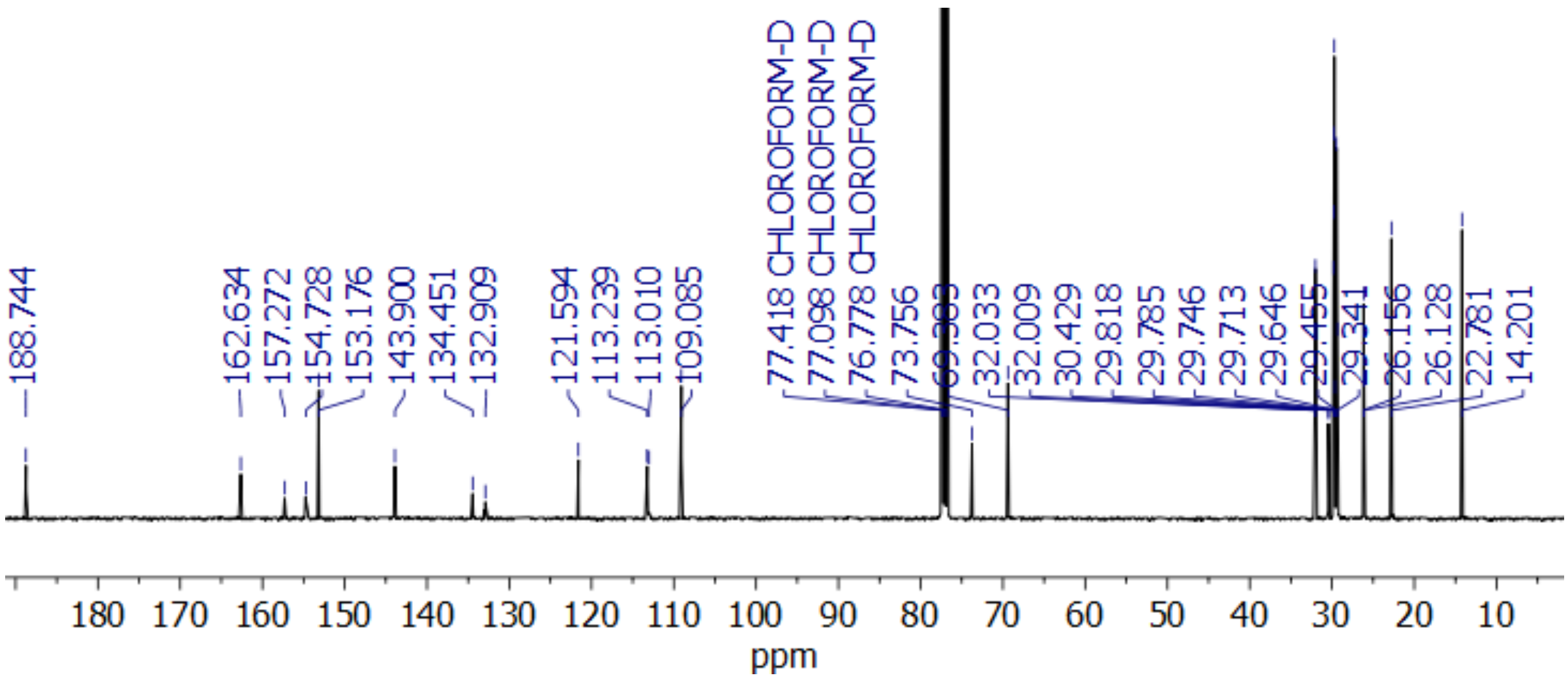

Figure S3.2. ^{13}C NMR spectrum ($CDCl_3$) of **A1**.

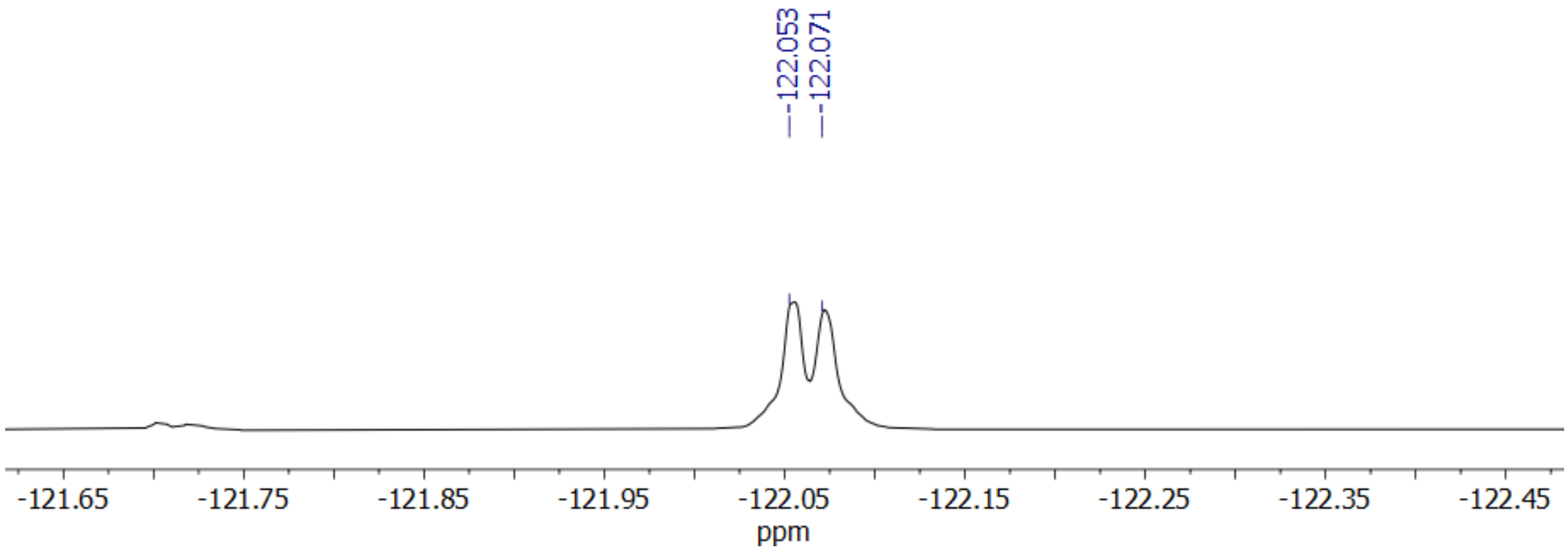

Figure S3.3. ^{19}F NMR spectrum ($CDCl_3$) of **A1**.

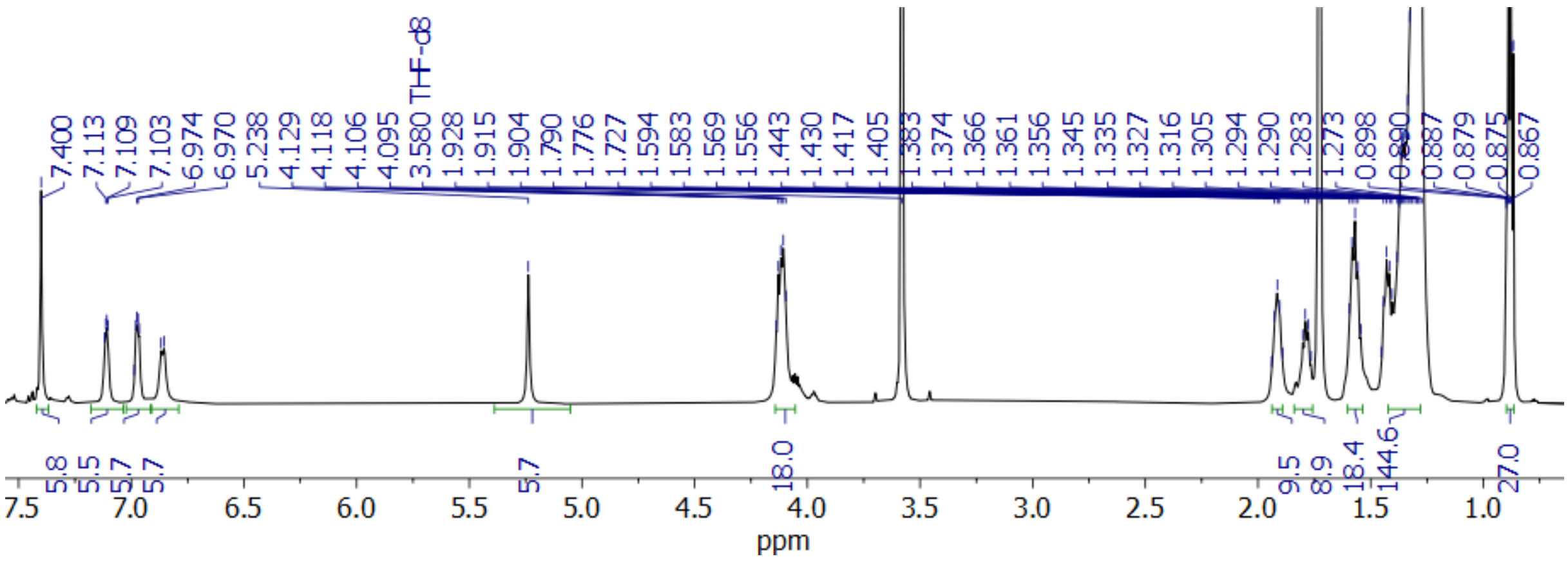

Figure S3.4. ^{1}H NMR spectrum (THF-d_8) of **NRos1**.

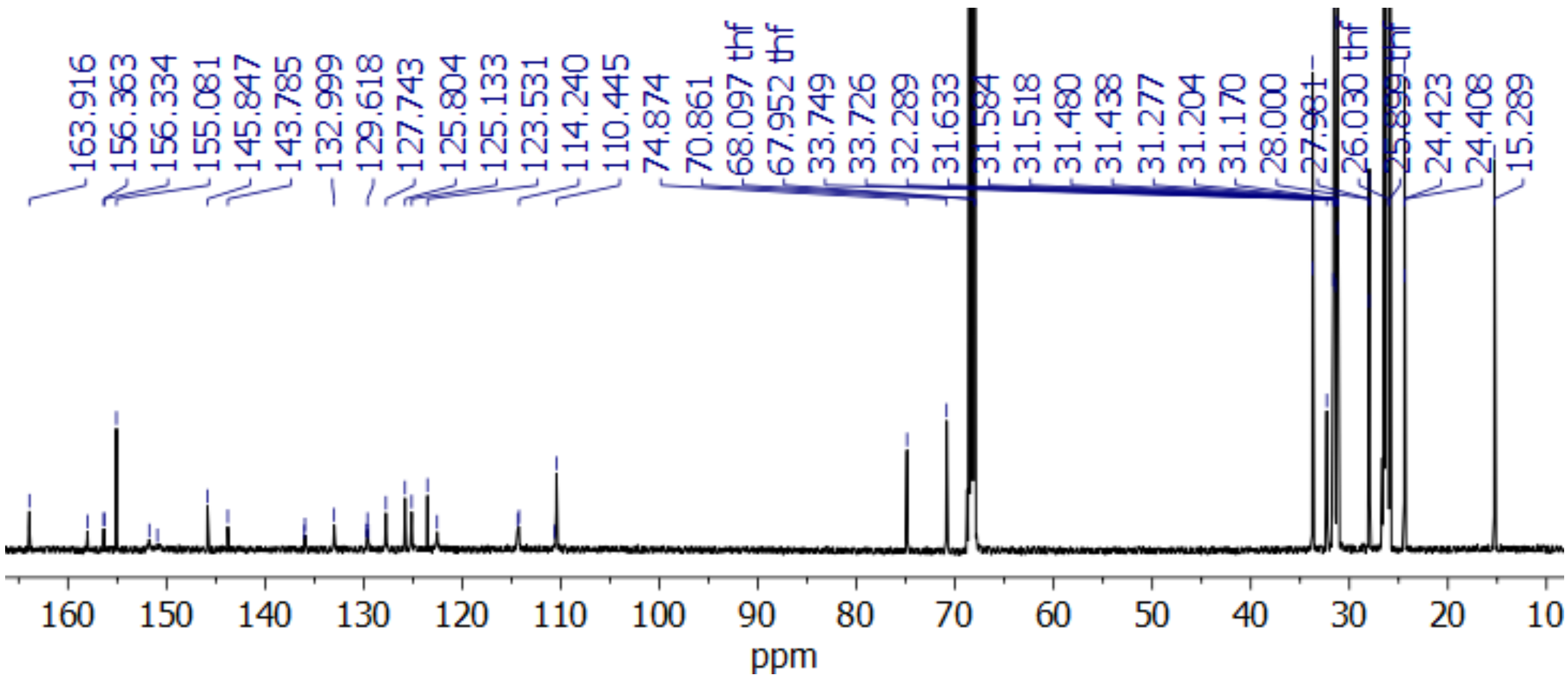

Figure S3.5. ^{13}C NMR spectrum (THF-d_8) of **NRos1**.

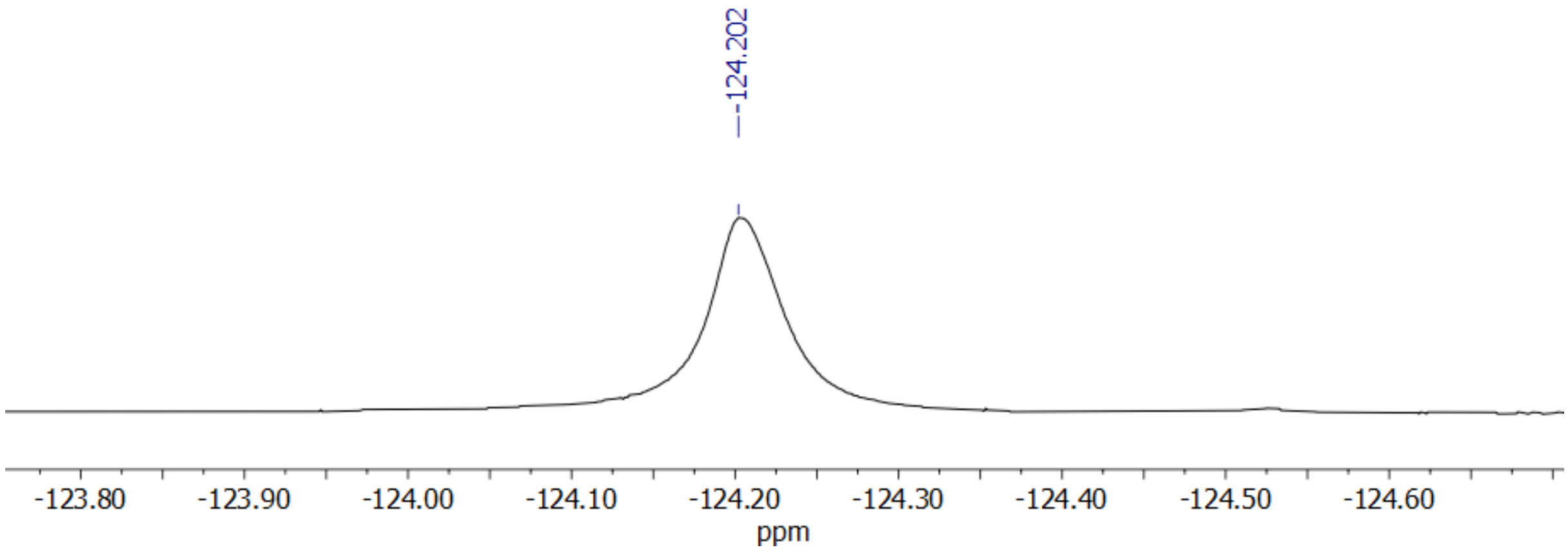

Figure S3.6. ^{19}F NMR spectrum (THF-d_8) of **NRos1**.

4. Mass Spectra

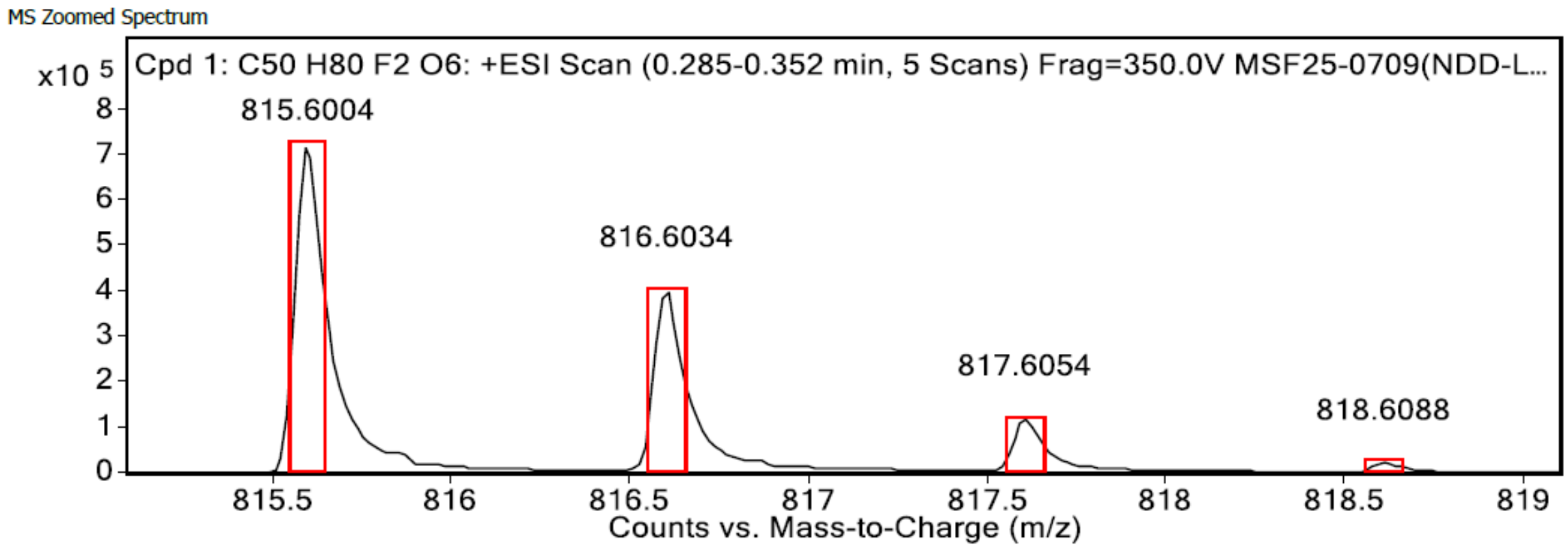

MS Spectrum Peak List

Obs. m/z	Calc. m/z	Charge	Abundance	Formula	Ion Species	Tgt Mass Error (ppm)
815.6004	815.5996	1	728660	C50H80F2O6	(M+H)+	-1.06
816.6034	816.6030	1	402346	C50H80F2O6	(M+H)+	-0.54
817.6054	817.6062	1	119025	C50H80F2O6	(M+H)+	0.94
818.6088	818.6093	1	21066	C50H80F2O6	(M+H)+	0.53
819.6112	819.6123	1	3911	C50H80F2O6	(M+H)+	1.34

--- End Of Report ---

Figure S4.1. HRMS of **A1** (ESI-TOF, positive mode, acetonitrile).

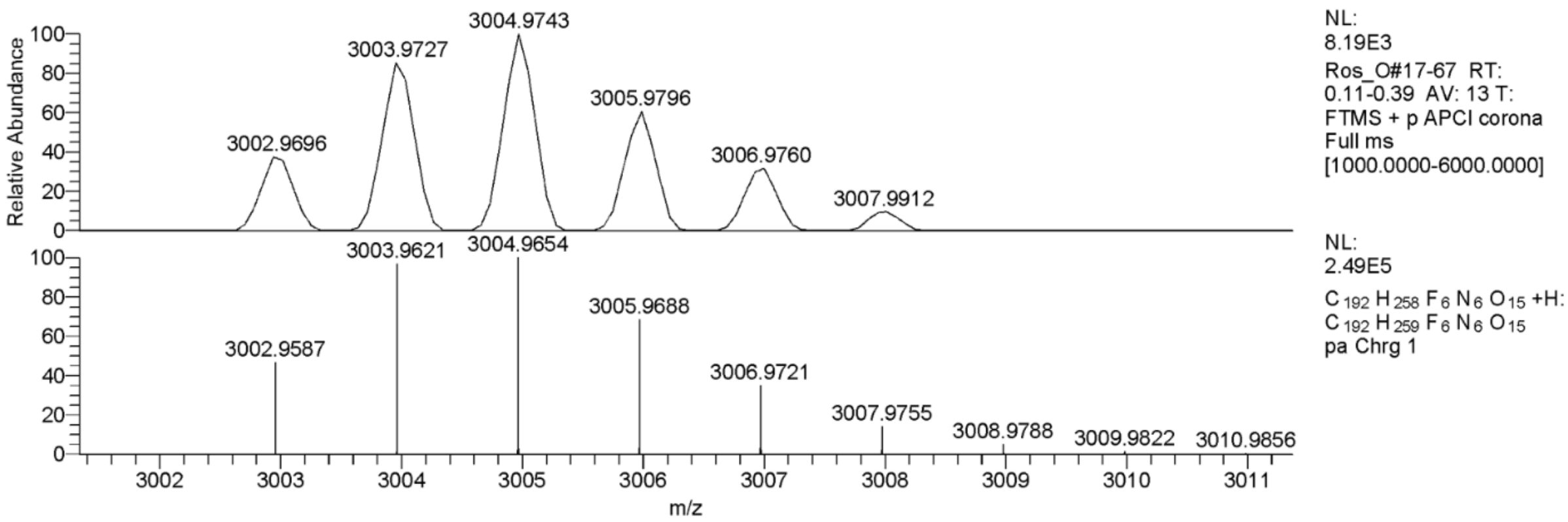

Figure S4.2. HRMS of **NRos1** (FT-APCI-MS, positive mode).

5. UV-Vis Spectra

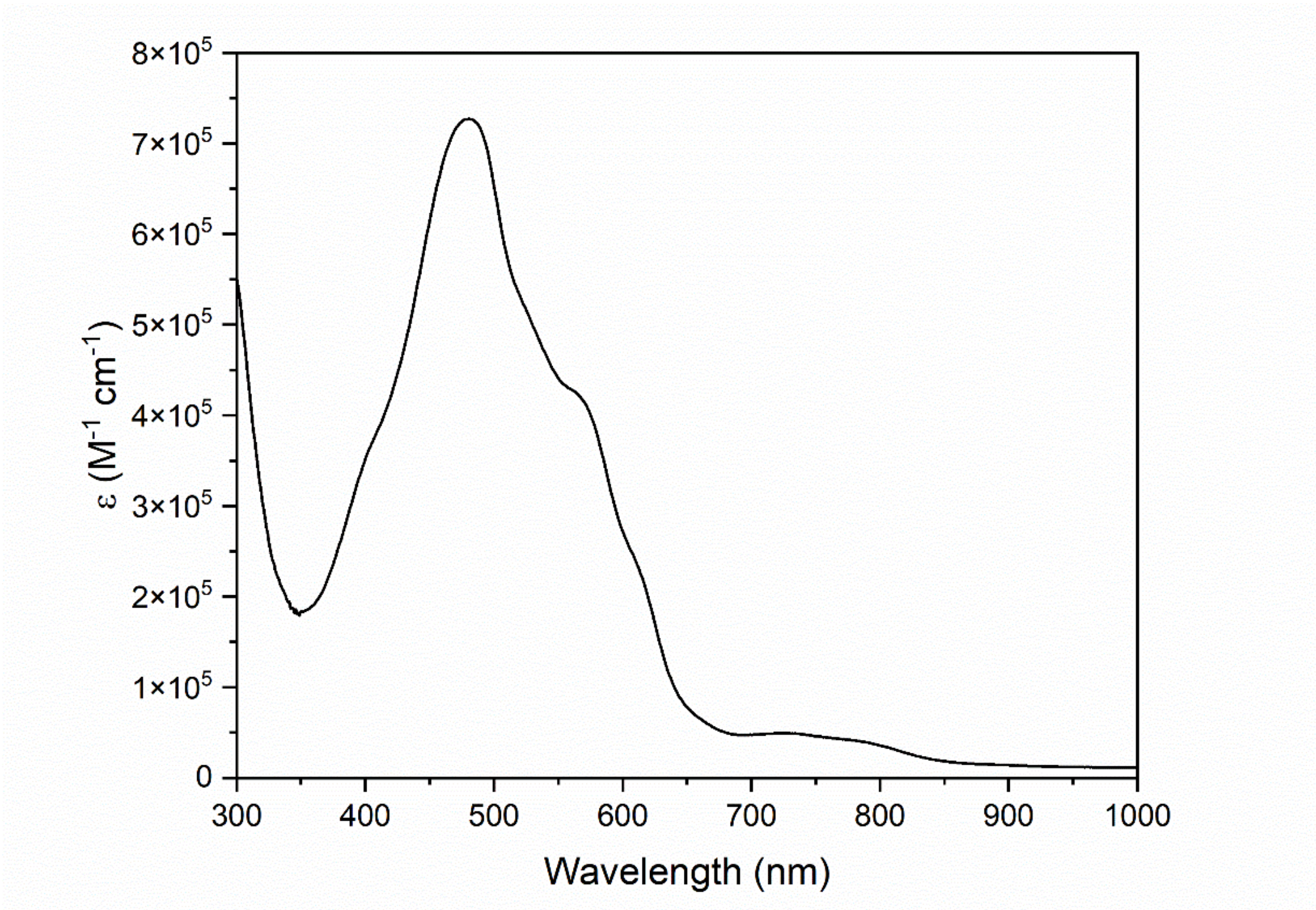

Figure S5.1. UV-vis absorption spectrum of **NRos1** (6 μM) in DCM solution.

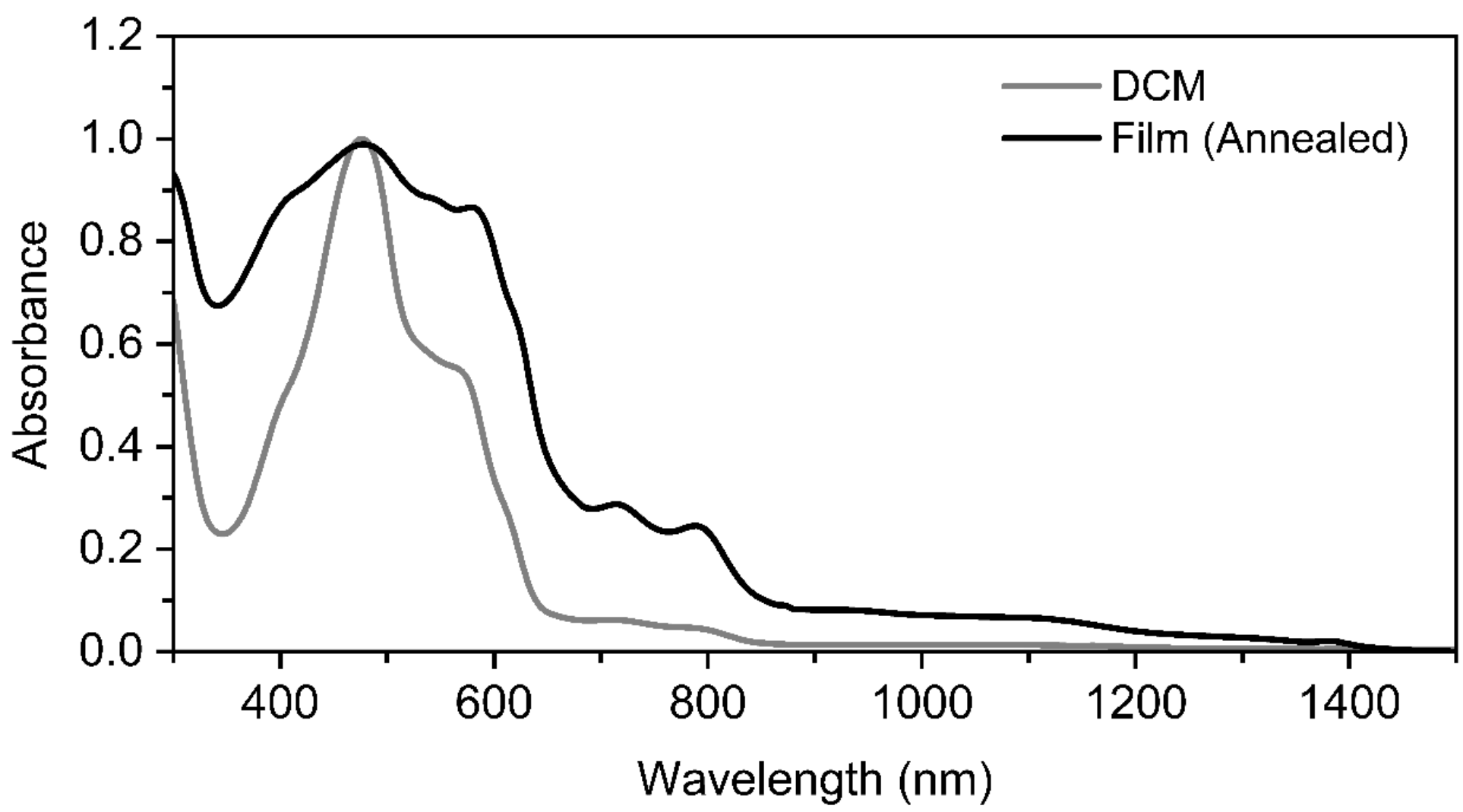

Figure S5.2. Normalized UV-vis spectra of **NRos1** in DCM solution (gray) and as a film (black). Films were formed by drop-casting 2.5 mg/mL solution in CH_2Cl_2 over a quartz substrate.

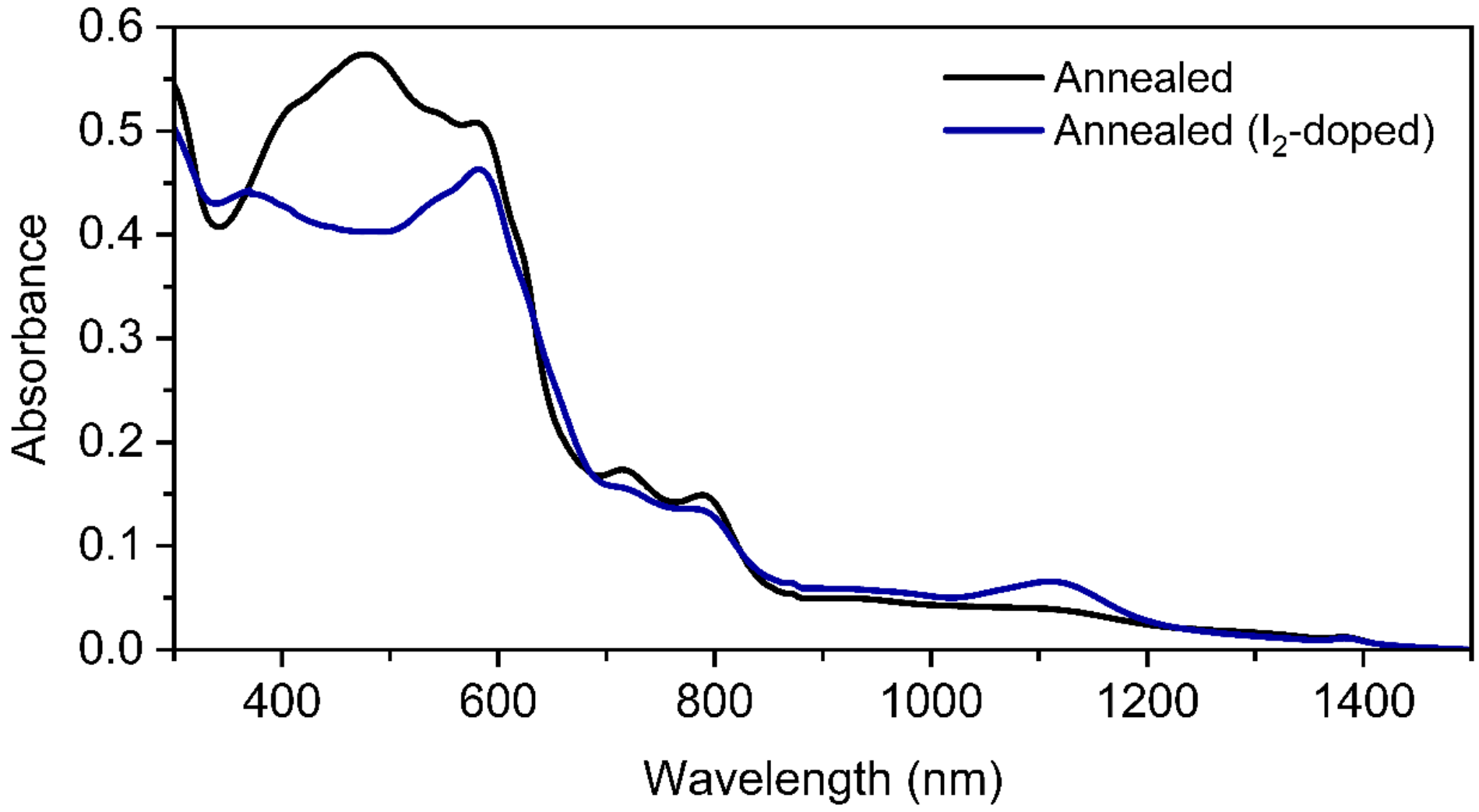

Figure S5.3. UV-vis spectral changes of **NRos1** as a film upon doping with I_2.

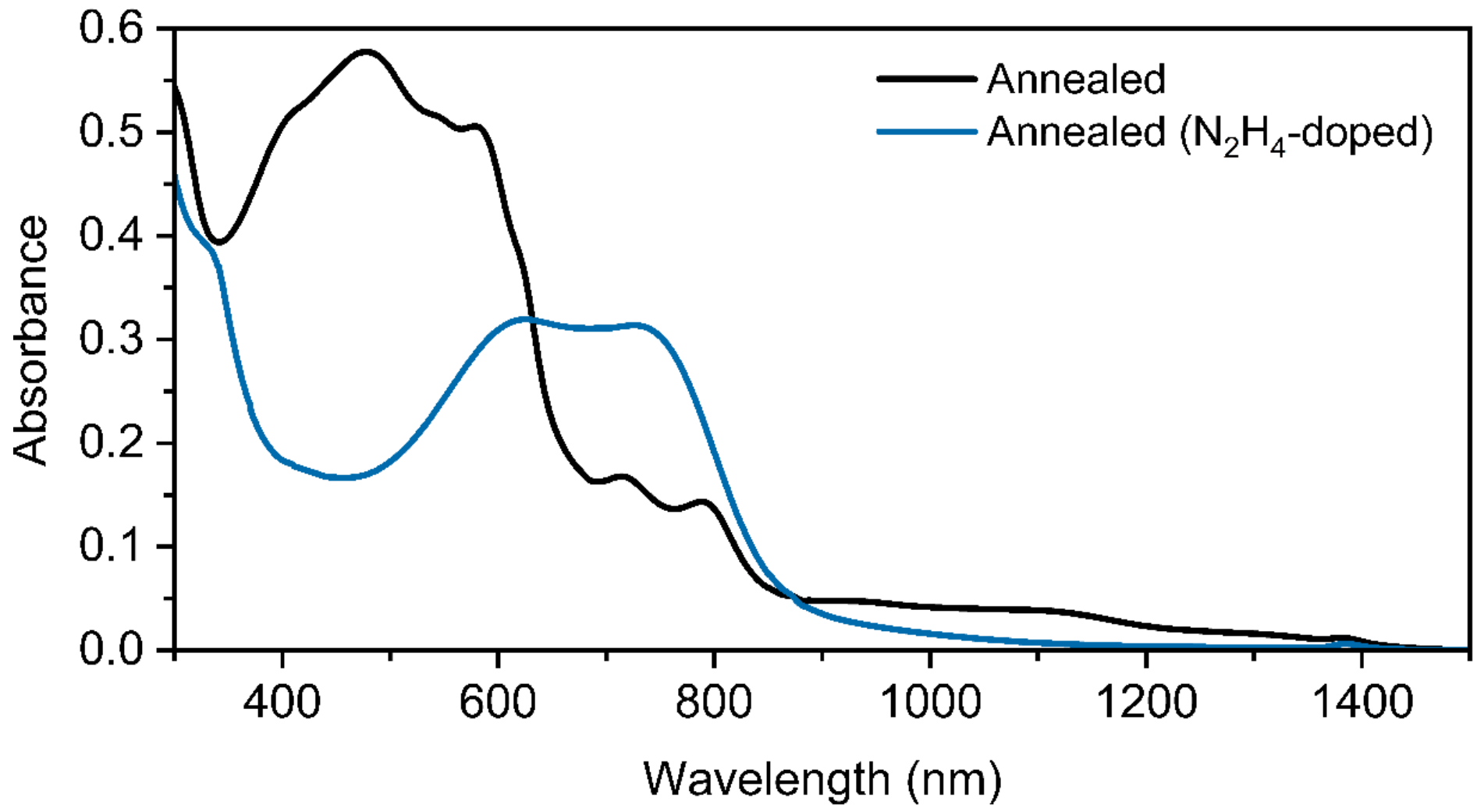

Figure S5.4. UV-vis spectral changes of **NRos1** as a film upon doping with hydrazine (N_2H_4) vapor. To accelerate the putative doping process, the film was placed in a sealed vial with N_2H_4•H_2O and heated at 50 °C for 30 min.

6. Electrochemistry and Spectroelectrochemistry

Cyclic voltammetry (CV) was carried out at 298K using a BAS ALS600D electrochemical analyzer paired with a homemade three-electrode glass cell consisting of a glassy carbon working electrode, a platinum wire auxiliary electrode, and an Ag/AgCl reference electrode which was separated from the bulk *via* a fritted glass bridge of low porosity filled with the corresponding solvent/supporting electrolyte. High purity argon gas was used to deaerate solutions prior to measurements. The inert environment was then maintained by flowing Ar over the solution during each potential sweep. Ferrocene (Fc) was used as an internal standard, and the reported potentials were corrected to the Fc/Fc^+ couple which was measured separately under the same solution conditions.

Thin-layer UV-Vis-NIR spectroelectrochemical (SEC) experiments were carried out at room temperature using a quartz cell (*b* = 0.05 cm) purchased from BAS paired with a homemade light transparent platinum net-working electrode, a platinum wire counter electrode and an Ag/AgCl reference electrode which was separated from the bulk *via* a fritted glass bridge of low porosity filled with the solvent/supporting electrolyte mixture. The potential dependent spectral changes between 300 and 1500 nm and thin-layer cyclic voltammograms (TLCV) were obtained *via* a potential sweep of the thin-layer cell (0.8 mV/s) to ensure the spectral change was completely reversible.

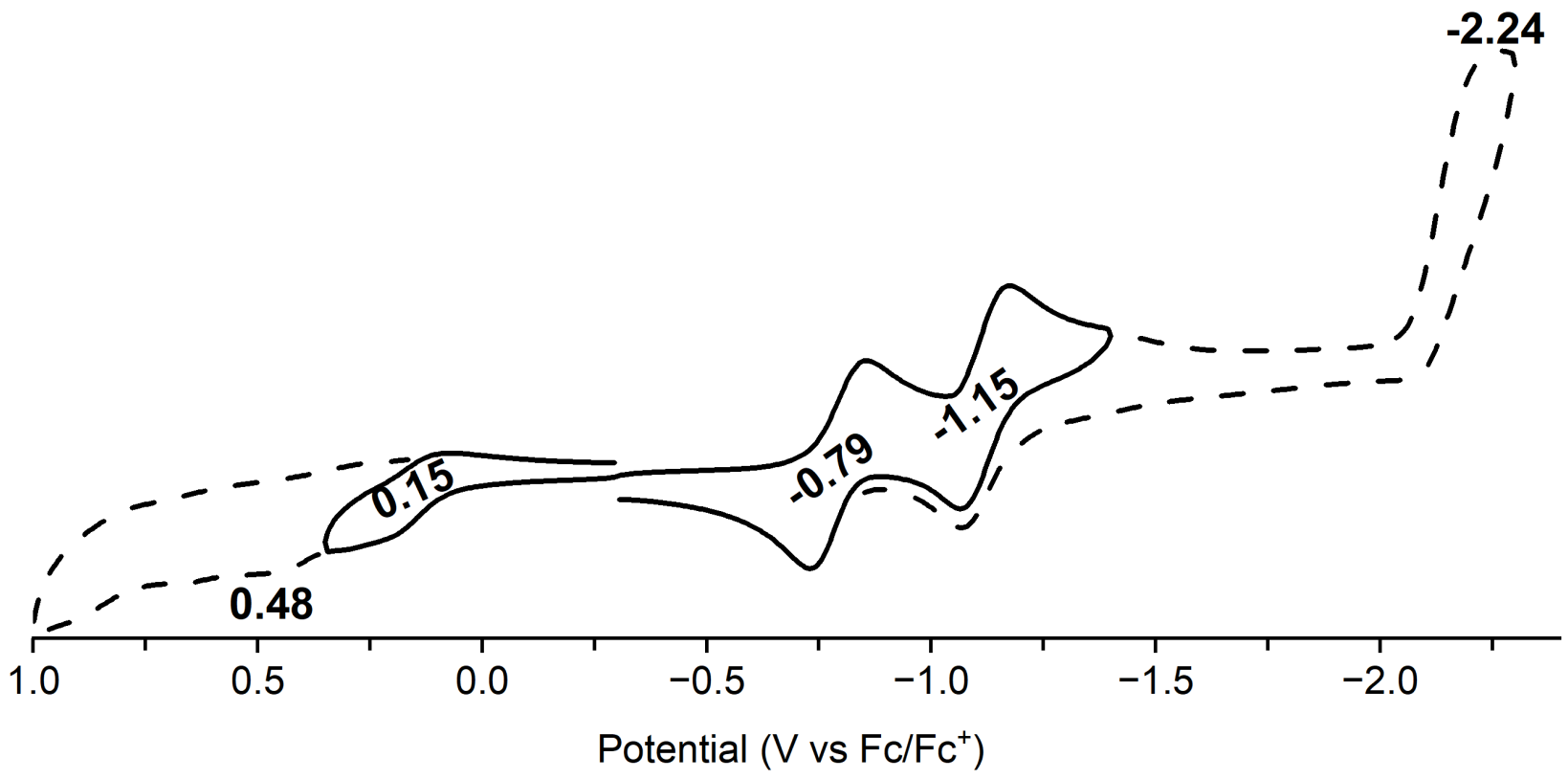

Figure S6.1. Cyclic voltammogram of the investigated **NRos1** in CH_2Cl_2 containing 0.1 M $TBAPF_6$. Scan rate = 50 mV/s.

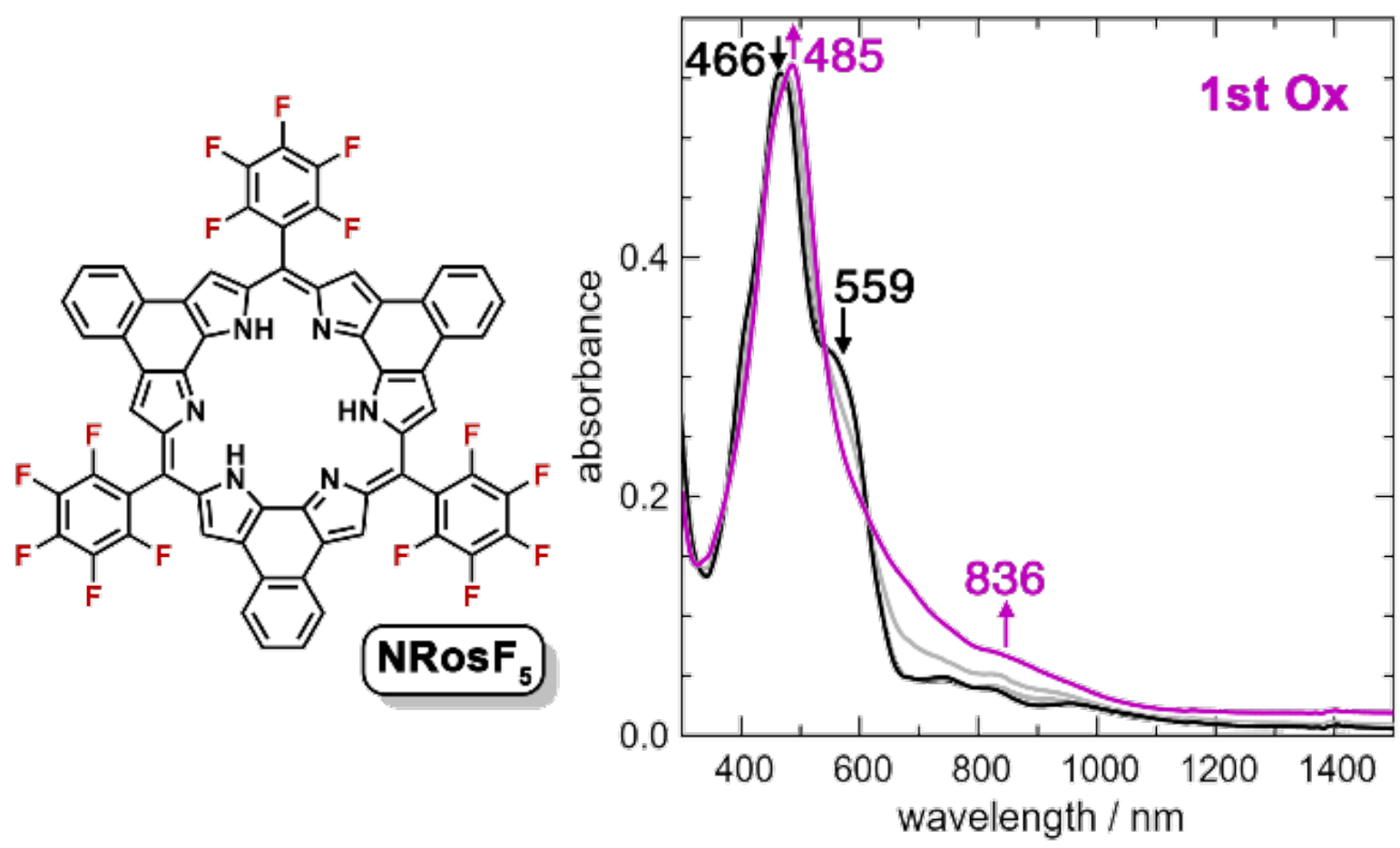

Figure S6.2. Potential dependent thin-layer UV-vis spectral changes of **NRosF$_5$** during the first and second controlled potential reductions and first oxidations in DCM containing 0.1 M $TBAPF_6$.

7. Computational Studies

All reported structures were optimized at the GFN2-xTB level[3] in the gas phase. Dimeric structures were also optimized by DFT to assess the discrepancies with the above-mentioned method. The DFT calculations were conducted using the B3LYP functional[4] and the standard 6-31G(d) basis set. All of the calculations were carried out using the methods implemented in the Gaussian 16 package.[5] As the DFT calculations proved concordant with those optimized at

the GFN2-xTB level, a decision was made to complete the study with GFN2-xTB in order to reduce computational costs.

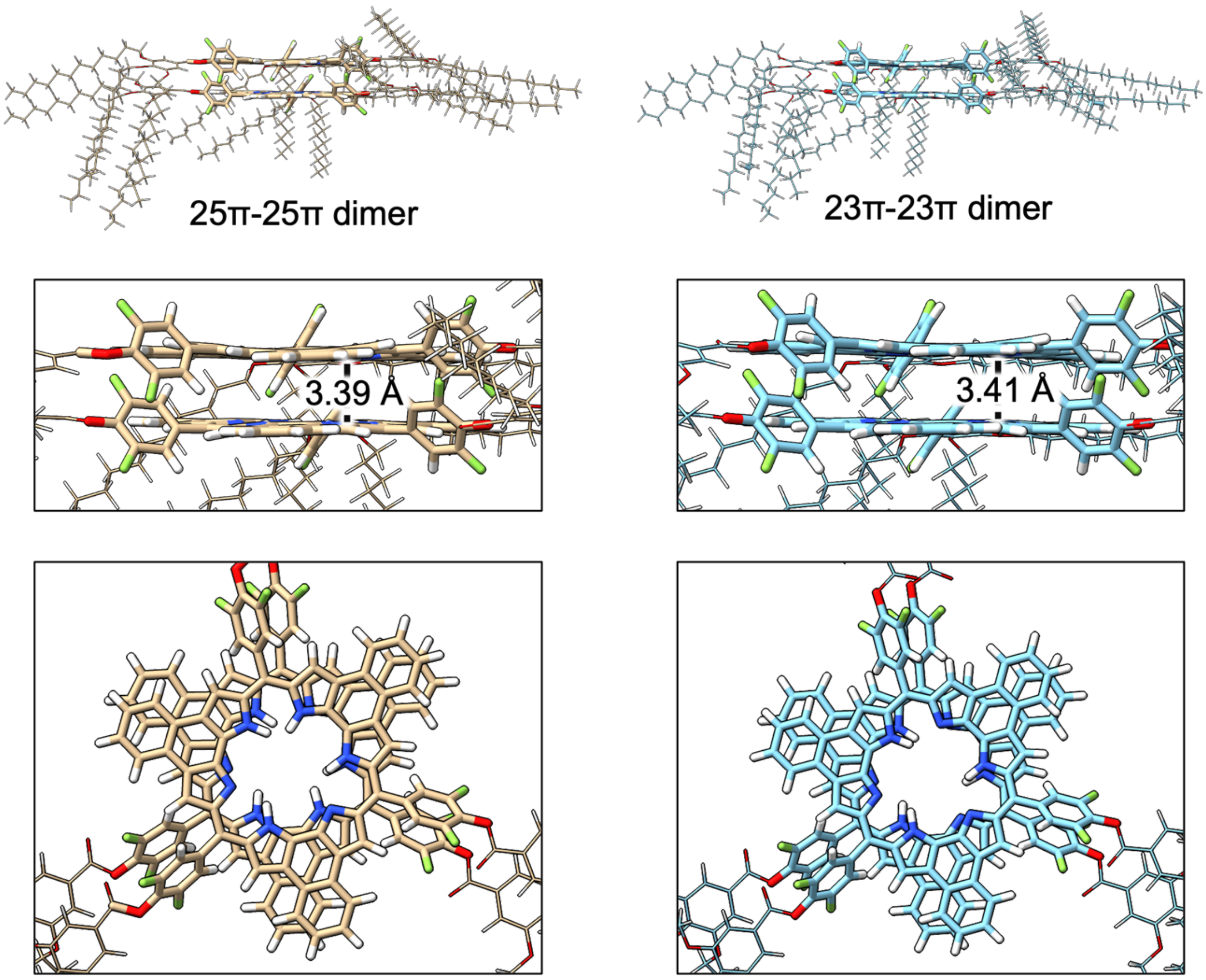

Figure S7.1. Top and side view of the GFN2-xTB-optimized geometry calculated for a supramolecular n-doped (25π-25π) and p-doped (23π-23π) dimer of **NRos1**.

8. Differential Scanning Calorimetry

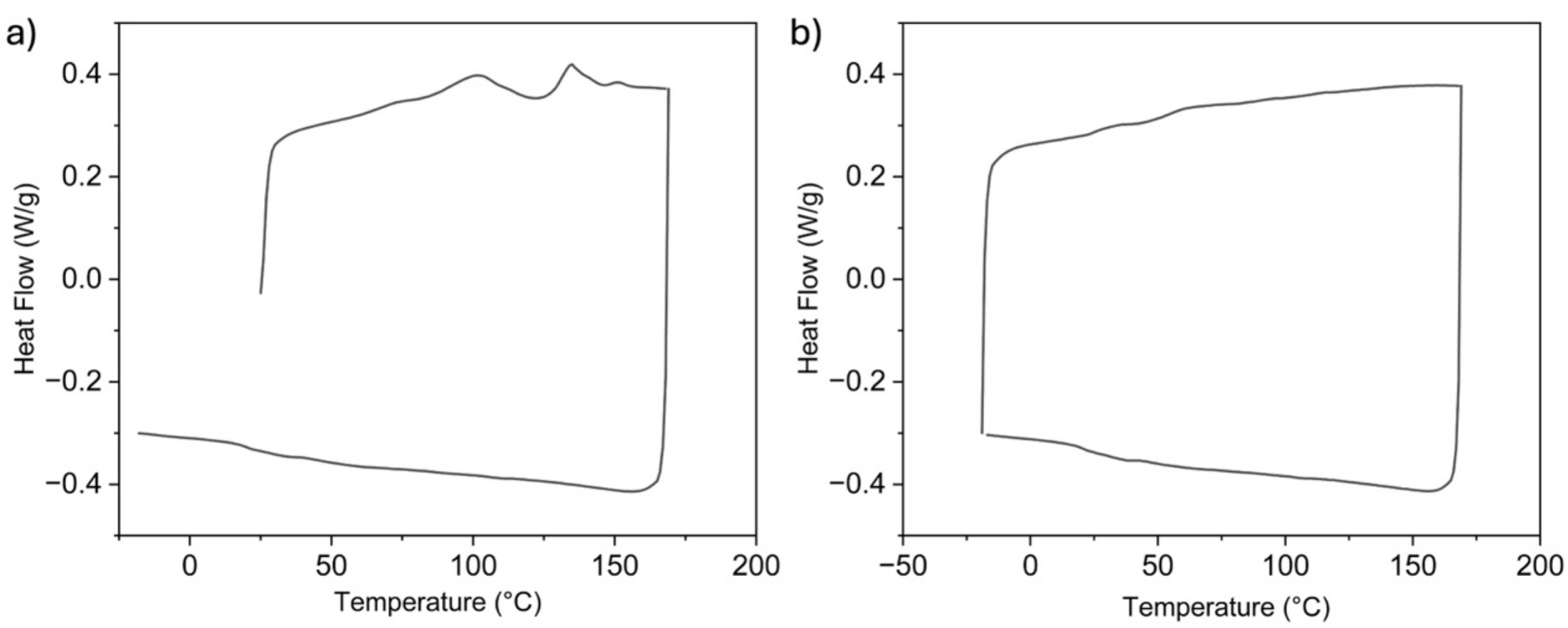

Figure S8.1. (a) First and (b) second heating-cooling thermograms recorded for **NRos1** at a rate of 10ºC/min.

9. X-ray Diffraction Studies

d_{obs}[a] (Å)	d_{calc}[b] (Å)	*h k*[c]	Lattice parameters[d]
32.9	32.9	1 1	*a*= 40.9 Å;
27.8	27.8	0 2	*b*= 55.6 Å
21.1	20.4	2 0	*c*= 3.8 Å
16.2	16.4	2 2	*Z* = 1
14.3	13.9	0 4	*ρ*= 1.14 g/cm^3
10.3	10.2	4 0	
9.4	9.4	4 2	
4.1 (br)		alkyl chains	
3.8 (br)		stacking	

[a] *E*xperimental *d* spacings obtained from XRD.

[b] *C*alculated *d* spacings for a rectangular columnar lattice using $d_{hk} = 1/\sqrt{h^2/a^2 + k^2/b^2}$

[c] Miller indices.

[d] Lattice parameters of the Col$_r$ phase: *a*= $1/\sqrt{1/d_{11}^2 - 1/(2 \cdot d_{02})^2}$; $b = 2 \cdot d_{02}$; *c*= mean stacking distance; *Z*= number of molecules per disk; calculated density value (ρ) = $(M/N_A)/(a \cdot b \cdot c \cdot 10^{-24}/Z)$, where *M* is the molecular mass, N_A Avogadro's number[6].

Table S1. Observed and calculated *d*-spacings for a thin film of **NRos1** analyzed by X-ray diffraction (XRD) at room temperature.

10. Flash-Photolysis Time-Resolved Microwave Conductivity (FP-TRMC)

TRMC measurements were performed on thin-films of **NRos1** using 355 nm pulsed photo-injection produced by third-harmonic generation (THG, λ = 355 nm) with a photon density of 9.1 × 10^{15} photons cm^{-2} from a Spectra-Physics INDI-HG Nd/YAG laser, operated with the pulse duration of 5−8 ns and 10 Hz. To assure the sensitivity and dynamic range of the microwave monitoring, the sample was inserted into an X-band microwave cavity resonating at the TE_{102} mode with *Q*~2500 at ca. ~9.1 GHz from a Gunn diode oscillator or from a signal generator (Rohde & Schwarz SMF100A). The resonant frequency and microwave power were set at ~9.1 GHz and 3 mW, respectively and the reflected microwave from the cavity was amplified and recorded with a MDO3024 oscilloscope and converted directly into the electric conductivity transients. The response time of $\sigma(t)$ is defined by the microwave cavity and proved to be ~ 50 ns. which is large in comparison with the other time constants, such as thoat of the Gunn diode, the laser pulse duration, and the monitoring amplifier system.

The monitored differential $\sigma(t)$ reflects mobility of the charge carriers, μ the number density of charge carriers, and Δn by Ohm's law as:

$$\sigma(t) = e\Delta n(t)\mu$$

where e is an elementary charge. According to the spatial distribution of light absorbed in the materials, the number of transient charge carriers: $\Delta n(t)$ is represented by photo-ionization quantum efficiency of $\mathrm{f}(t)$, which is plotted in Figure 4a. The maximum values of were determined by averaging 50 data points around time zero. This averaging window was chosen based on the instrumental response time of 50 ns for our setup. n- and p-doping was performed

by exposure to excess N_2H_4 or I_2 vapor for 30 min in a sealed vial at 35 °C and room temperature, respectively. This exposure time was found to be the minimum required for the photoconductivity to reach saturation.

11. Supporting References

[1] Roznyatovskiy, V., Lynch, V. and Sessler, J. L., *Org. Lett.* 2010*,* **12**, 4424–4427.

[2] M. Ishida, S.-J. Kim, C. Preihs, K. Ohkubo, J. M. Lim, B. S. Lee, J. S. Park, V. M. Lynch, V. V. Roznyatovskiy, T. Sarma, P. K. Panda, C.-H. Lee, S. Fukuzumi, D. Kim, J. L. Sessler, *Nat. Chem.* **2013**, 5, 15–20.

[3] C. Bannwarth, S. Ehlert, S. Grimme, *J. Chem. Theory Comput.* 2019, **15**, 1652–1671

[4] Gaussian 16, Revision C.01; Frisch, M. J.; Trucks, G. W.; Schlegel, H. B.; Scuseria, G. E.; Robb, M. A.; Cheeseman, J. R.; Scalmani, G.; Barone, V.; Petersson, G. A.; Nakatsuji, H.; Li, X.; Caricato, M.; Marenich, A. V.; Bloino, J.; Janesko, B. G.; Gomperts, R.; Mennucci, B.; Hratchian, H. P.; Ortiz, J. V.; Izmaylov, A. F.; Sonnenberg, J. L.; Williams-Young, D.; Ding, F.; Lipparini, F.; Egidi, F.; Goings, J.; Peng, B.; Petrone, A.; Henderson, T.; Ranasinghe, D.; Zakrzewski, V. G.; Gao, J.; Rega, N.; Zheng, G.; Liang, W.; Hada, M.; Ehara, M.; Toyota, K.; Fukuda, R.; Hasegawa, J.; Ishida, M.; Nakajima, T.; Honda, Y.; Kitao, O.; Nakai, H.; Vreven, T.; Throssell, K.; Montgomery, J. A., Jr.; Peralta, J. E.; Ogliaro, F.; Bearpark, M. J.; Heyd, J. J.; Brothers, E. N.; Kudin, K. N.; Staroverov, V. N.; Keith, T. A.; Kobayashi, R.; Normand, J.; Raghavachari, K.; Rendell, A. P.; Burant, J. C.; Iyengar, S. S.; Tomasi, J.; Cossi, M.; Millam, J. M.; Klene, M.; Adamo, C.; Cammi, R.; Ochterski, J. W.; Martin, R. L.; Morokuma, K.; Farkas, O.; Foresman, J. B.; Fox, D. J. Gaussian, Inc., Wallingford CT, 2016

[5] Y. Zhao, D. G. Truhlar, Theor. Chem. Acc., 2008, 120, 215.

[6] F. Vera, R. M. Tejedor, P. Romero, J. Barbera, M. B. Ros, J. L. Serrano, T. Sierra, Angew. Chem. Int. Ed. 2007, 46, 1873–1877.
